# The effectiveness of coronary computed tomography angiography and functional testing for the diagnosis of obstructive coronary artery disease: results from the individual patient data Collaborative Meta-Analysis of Cardiac CT (COME-CCT)

**DOI:** 10.1186/s13244-024-01702-y

**Published:** 2024-08-14

**Authors:** Peter Schlattmann, Viktoria Wieske, Keno K. Bressem, Theresa Götz, Georg M. Schuetz, Daniele Andreini, Gianluca Pontone, Hatem Alkadhi, Jörg Hausleiter, Elke Zimmermann, Bernhard Gerber, Abbas A. Shabestari, Matthijs F. L. Meijs, Akira Sato, Kristian A. Øvrehus, Shona M. M. Jenkins, Juhani Knuuti, Ashraf Hamdan, Bjørn A. Halvorsen, Vladimir Mendoza-Rodriguez, Johannes Rixe, Yung-Liang Wan, Christoph Langer, Sebastian Leschka, Eugenio Martuscelli, Said Ghostine, Jean-Claude Tardif, Alejandra Rodríguez Sánchez, Robert Haase, Marc Dewey

**Affiliations:** 1 Institute of Medical Statistics, Computer Sciences, and Data Science, University Hospital of Friedrich Schiller University Jena, Jena, Germany; 2 Department of Radiology, Charité—Universitätsmedizin Berlin, Charitéplatz 1, 10117 Berlin, Germany; 3 Centro Cardiologico Monzino, IRCCS, Milan, Italy; 4 Institute of Diagnostic and Interventional Radiology University Hospital Zurich, Zurich, Switzerland; 5 Maximilians-University of Munich, Munich, Germany; 6 Department of Cardiology, Clinique Universitaire St Luc, Institut de Recherche Clinique et Expérimentale, Brussels, Belgium; 7 Modarres Hospital, Shahid Beheshti University of Medical Sciences, Tehran, Iran; 8 Department of Cardiology, University Medical Centre Utrecht, Utrecht, The Netherlands; 9 Cardiovascular Division, Faculty of Medicine, University of Tsukuba, Tsukuba, Japan; 10 Department of Cardiology, Odense University Hospital, Odense, Denmark; 11 Glasgow Royal Infirmary and Stobhill Hospital, Glasgow, UK; 12 Turku University Hospital and University of Turku, Turku, Finland; 13 Department of Cardiovascular Imaging, Department of Cardiology, Rabin Medical Center, Sackler Faculty of Medicine, Tel-Aviv University, Tel-Aviv, Israel; 14 Department of Cardiology, Ostfold Hospital Trust, Grålum, Norway; 15 Department of Cardiology, National Institute of Cardiology and Cardiovascular Surgery, Havana, Cuba; 16 Department of Cardiology and Electrophysiology, Jung Stilling Hospital Siegen, Siegen, Germany; 17 Medical Imaging and Radiological Sciences, College of Medicine, Chang Gung University, Chang Gung Memorial Hospital at Linkou, Taoyaun City, Taiwan; 18 Kardiologisch-Angiologische Praxis, Herzzentrum Bremen, Bremen, Germany; 19 Department of Radiology, Kantonsspital St Gallen, St Gallen, Switzerland; 20 Department of Internal Medicine, University of Rome Tor Vergata, Rome, Italy; 21 Department of Cardiology, Centre Chirurgical Marie Lannelongue, Le Plessis Robinson, France; 22 Montreal Heart Institute, Université de Montréal, Montréal, QC Canada; 23 Berlin Institute of Health, Berlin, Germany; 24 DZHK (German Centre for Cardiovascular Research), Partner Site Berlin, Berlin, Germany

**Keywords:** Computed tomography angiography, Functional stress testing, Exercise-ECG, Single-photon emission computed tomography, Diagnostic accuracy

## Abstract

**Aim:**

To determine the effectiveness of functional stress testing and computed tomography angiography (CTA) for diagnosis of obstructive coronary artery disease (CAD).

**Methods and results:**

Two-thousand nine-hundred twenty symptomatic stable chest pain patients were included in the international Collaborative Meta-Analysis of Cardiac CT consortium to compare CTA with exercise electrocardiography (exercise-ECG) and single-photon emission computed tomography (SPECT) for diagnosis of CAD defined as ≥ 50% diameter stenosis by invasive coronary angiography (ICA) as reference standard. Generalised linear mixed models were used for calculating the diagnostic accuracy of each diagnostic test including non-diagnostic results as dependent variables in a logistic regression model with random intercepts and slopes. Covariates were the reference standard ICA, the type of diagnostic method, and their interactions. CTA showed significantly better diagnostic performance (*p* < 0.0001) with a sensitivity of 94.6% (95% CI 92.7–96) and a specificity of 76.3% (72.2–80) compared to exercise-ECG with 54.9% (47.9–61.7) and 60.9% (53.4–66.3), SPECT with 72.9% (65–79.6) and 44.9% (36.8–53.4), respectively. The positive predictive value of CTA was ≥ 50% in patients with a clinical pretest probability of 10% or more while this was the case for ECG and SPECT at pretest probabilities of ≥ 40 and 28%. CTA reliably excluded obstructive CAD with a post-test probability of below 15% in patients with a pretest probability of up to 74%.

**Conclusion:**

In patients with stable chest pain, CTA is more effective than functional testing for the diagnosis as well as for reliable exclusion of obstructive CAD. CTA should become widely adopted in patients with intermediate pretest probability.

**Systematic review registration:**

PROSPERO Database for Systematic Reviews—CRD42012002780.

**Critical relevance statement:**

In symptomatic stable chest pain patients, coronary CTA is more effective than functional testing for diagnosis and reliable exclusion of obstructive CAD in intermediate pretest probability of CAD.

**Key Points:**

- Coronary computed tomography angiography showed significantly better diagnostic performance (*p* < 0.0001) for diagnosis of coronary artery disease compared to exercise-ECG and SPECT.
- The positive predictive value of coronary computed tomography angiography was ≥ 50% in patients with a clinical pretest probability of at least 10%, for ECG ≥ 40%, and for SPECT 28%.
- Coronary computed tomography angiography reliably excluded obstructive coronary artery disease with a post-test probability of below 15% in patients with a pretest probability of up to 74%.

**Graphical Abstract:**

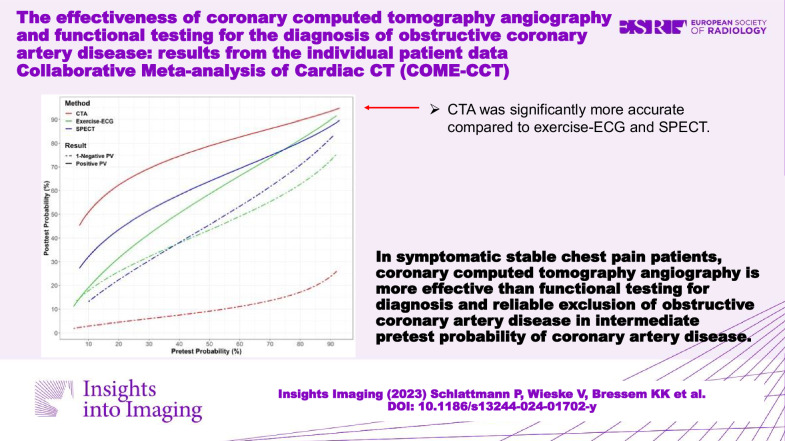

## Introduction

Coronary computed tomography angiography (CTA) is increasingly used to diagnose coronary artery disease (CAD). Indeed, clinical guideline 95 of the National Institute for Health and Care Excellence with chest pain of recent onset recommends CTA as the first diagnostic test in all patients with possible angina [1]. Functional stress testing, including exercise electrocardiography (exercise-ECG) or single-photon emission computed tomography (SPECT), is recommended in uncertainty about whether chest pain is caused by myocardial ischaemia in patients with known CAD. In contrast, the recent ESC guideline on chronic coronary syndrome (CCS) recommends coronary CTA as the first-line diagnostic imaging test with a low pretest probability for CCS, whereas functional cardiac imaging is recommended in patients having a high pretest probability for CCS [2]. The ISCHEMIA trial showed that an invasive interventional strategy was not superior to a conservative strategy in patients with stable chest pain and test-based ischaemia [3].

Results of the SCOT-HEART trial showed a significant reduction of fatal and non-fatal myocardial infarction by CTA compared with diagnostic standard of care in patients with recent onset stable chest pain [4]. However, there is a lack of large diagnostic comparison studies of CTA for coronary stenosis evaluation with functional stress testing for ischaemia evaluation for the detection of obstructive CAD. Previous investigations have suggested that coronary CT may have higher sensitivity and specificity than functional stress testing for the detection of anatomically defined CAD with invasive coronary angiography (ICA) as the reference standard [5–7]. Within the Collaborative Meta-Analysis of Cardiac CT (COME-CCT) [8] of patients with symptomatic stable chest pain, we compared the effectiveness of functional stress testing using exercise-ECG or SPECT with CTA for diagnosis of CAD using ICA as the reference standard. Further, the association of non-invasive diagnostic tests and pretest probability was assessed for evaluation of the ability to exclude obstructive CAD.

## Methods

### Patients

Seven-thousand eight-hundred thirteen patients with stable chest pain and suspected CAD were included in the COME-CCT Consortium with a clinical indication for ICA, who were also prospectively enrolled to undergo cardiac CT. The study protocol of the COME-CCT collaborators was previously published including detailed information on search strategy, inclusion, and exclusion criteria for this individual patient data (IPD) data meta-analysis [8]. Patients with stents or bypasses, unstable angina, and non-diagnostic were excluded as well as patients with incomplete information for pretest probability calculation. Data was available on the per-patient level. The study was prospectively registered in the PROSPERO Database for Systematic Reviews (CRD42012002780). Obstructive CAD was defined as at least diameter stenosis of ≥ 50% by ICA with 81% of patients receiving quantitative coronary analysis (QCA). Specifically important for the present subgroup analysis, studies were excluded if datasets did not include results on either exercise-ECG or SPECT for at least 5% of the patients. All participants gave written informed consent to participate in the local studies, which were approved by the local ethics committees of the participating centres. For quality assessment and comparability, an additional questionnaire regarding exercise-ECG and SPECT was sent to all participating sites. For this subanalysis, for those studies eligible for inclusion, patients with data on functional testing were included, but studies with < 5% of patients receiving functional testing with regard to the site cohort were excluded from further analysis to avoid inclusion bias.

### Statistical analysis

**Fig. 1:**
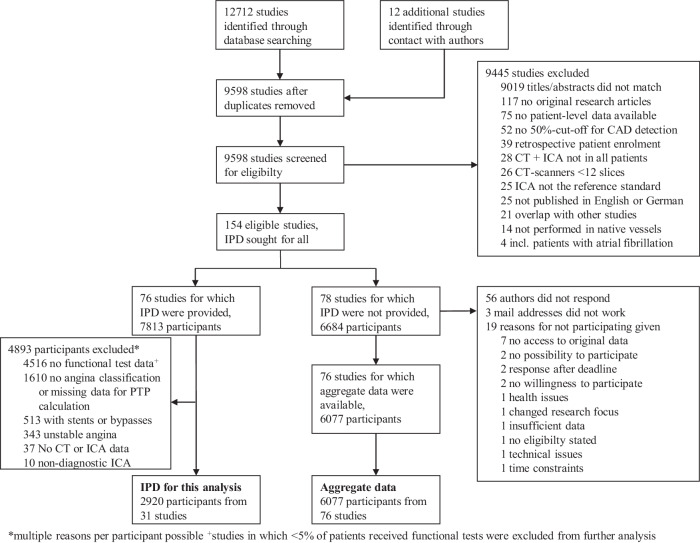
Flow chart for study selection. Part of the study flow referring to the COME-CCT main analysis paper as published by Haase et al [70]. In this subanalysis of the international COME-CCT Consortium, only patients with functional testing data were included, and studies for pooled analysis were only available if at least 5% of the patients of each of the 31 included studies received functional testing in order to avoid inclusion bias

Raw datasets were merged in an Excel spreadsheet and exported as comma-separated values for statistical analysis using “R” [9]. Continuous data are reported as mean (standard deviation (SD)) and categorical variables as percentages (absolute numbers). Diagnostic accuracy of all tests using obstructive CAD defined by ICA as the reference standard was modelled using generalised linear mixed models (GLMM), i.e. multivariable logistic regression model with a study-specific random intercept to take heterogeneity between studies into account [10] by extending the method suggested by Coughlin et al with random effects, which provides a one-step approach for a diagnostic IPD meta-analysis [11]. The current model is a univariate logistic regression model extended by incorporating a random effect for the study and a random slope for ICA results, respectively, which is equivalent to a bivariate generalised linear mixed model [12]. Based on this model using the test result as the dependent variable, mean logit sensitivity and specificity, the estimates of the between-study variability in logit sensitivity and specificity, and the covariance between them were estimated. These estimates quantify heterogeneity between studies and patients within studies and investigate the effect of covariates such as type of diagnostic procedure. Covariates were: the reference standard ICA and the type of non-invasive diagnostic method and their interactions. Post-test probabilities (positive (PPVs) and negative predictive values (NPVs)) of the respective diagnostic procedures for the presence of CAD as a function of the pretest probability of CAD were analysed by a generalised linear mixed model as described above. In a similar way, models were applied when studies with a high risk of bias were analysed in a sensitivity analysis. In another analysis, we compared the diagnostic accuracy of CT in the 2920 patients from studies with functional tests performed with 2412 patients who were included in studies in which no functional tests were performed (Fig. 1) applying the covariate test performed (yes/no). Using an intention-to-diagnose approach, we implemented the worst-case scenario in which non-diagnostic CTA results were considered false positive if ICA was negative and false negative if ICA was positive [13]. Clinical pretest probability was calculated using a validated prediction tool, which was an updated version of the Diamond and Forrester model [14, 15]. Clinical pretest probability was estimated based on patient age, gender, and clinical presentation. We also performed a statistical prediction for a new cohort following the ideas presented by Skrondal and Rabe-Hasketh [16].

Diagnostic performance was evaluated using a complete case analysis (basic generalised linear model) while the role of potential covariates was investigated using multiple imputations of missing data in patients who did not undergo functional test results in the original studies as a sensitivity analysis. Statistical analysis was performed with “R” (R-package lme4) [17]. For the reduction of missing data bias, multiple imputation was performed. Post-test probabilities were obtained with STATA 14 (packages GLLAMM, GLLAPRED). Cross-hair plots which show a scatter plot per-study sensitivity and false-positive rate with corresponding confidence intervals were produced with the R package mada [18, 19].

## Results

### Study characteristics

**Fig. 2:**
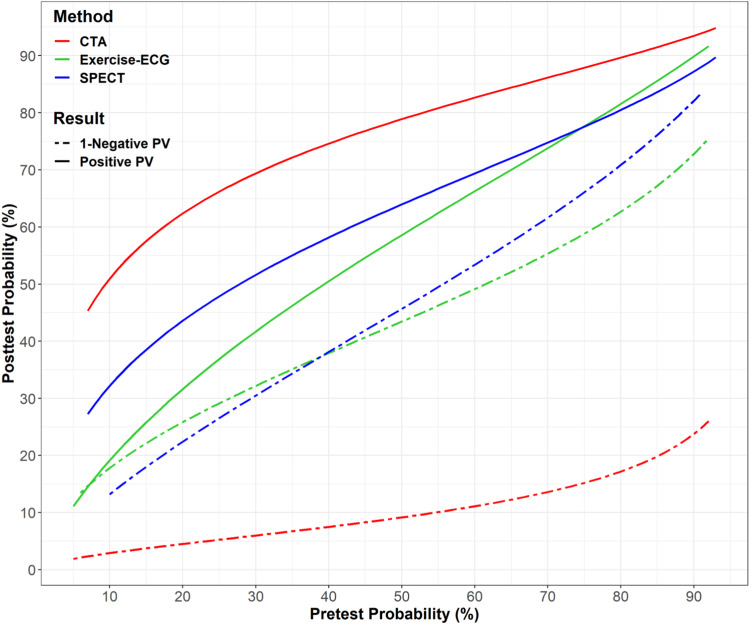
Analysis of diagnostic performance for CTA, Exercise-ECG, SPECT. The lines represent the positive and negative predictive values of CAD after a positive (solid lines) or negative (dashed lines) diagnostic test result for obstructive (obstructive) coronary artery disease defined as a patient with at least 50% coronary diameter stenosis. CTA was significantly more accurate than exercise-ECG and SPECT. Predictive values including 95% confidence intervals for all three tests are provided in Appendix Figs. 3–5

Pooled data on the per-patient level from 31 eligible studies with data from 2920 patients from 21 sites in 16 countries for analysis (Fig. 1) [5–7, 20–46]. Results of consensus reviewer judgments of the methodological quality of included studies regarding risk of bias and applicability can be found in the Appendix (Figs. 1 and 2). The risk of bias was high in eight studies and high applicability concerns were not present [7, 25, 30, 33, 37, 40, 41, 45]. Included participant data varied in size from 3 to 243 participants (mean (SD) of 91.2 (53.9)); 67% were male (Table 1). All patients included underwent clinically indicated ICA, (81% with QCA) as the reference standard for detection of obstructive CAD.

**Table 1:** Patient characteristics

Continuous characteristics									
	Overall			Women			Men		
	Mean (SD)	Range	Missings, %	Mean (SD)	Range	Missings, %	Mean (SD)	Range	Missings, %
Age	61.7 (10)	26–92	0	62.9 (10.2)	26.9–91	0	61.1 (9.9)	26–92	0
BMI	26.6 (3.7)	15.5–45.2	0.03	26.6 (4.3)	16.7–45.2	0.1	26.7 (3.4)	15.5–41.8	0
Pre-test probability	47.9% (22.2)	5–93%	0%	19.2%	5–77%	0%	55.7% (19.3)	15–93%	0%

Binary characteristics									
	Overall			Women			Men		
	*N*	Percentage	Missings, %	*N*	Percentage	Missings, %	*n*	Percentage	Missings, %
Total	2920		0	963	33	0	1957	67	0
Diabetes	582	19.9	0.03	159	16.5	0	423	21.6	0.05
Dyslipidaemia	1603	54.9	0.24	529	54.9	0.62	1074	54.9	0.05
Hypertension	1581	54.1	0	535	55.6	0	1046	53.4	0
Slightly obese (BMI 25–30)	1470	50.3	0.03	441	30	0.1	1029	70	0
Obesity class I (BMI 30–35)	413	14.1	0.03	139	33.7	0.1	274	66.3	0
Obesity class II or above (BMI > 35)	68	2.3	0.03	39	57.3	0.1	29	42.6	0
Family history of CAD	898	30.8	10.7	317	32.9	16.7	581	29.7	7.7
Active smoker	745	25.5	0.03	195	20.2	0.1	550	28.1	0
Former smoker	575	19.7	7.7	190	19.7	8.3	385	19.7	7.4

*BMI* body mass index, *CAD* coronary artery disease, *SD* standard deviation

### Imaging test characteristics

Fifty-three percent of the patients had an additional exercise-ECG (1540/2920), 37% had no functional test (1066/2920) and 18% had an additional SPECT (532/2920). Approximately 7% of patients underwent all three non-invasive tests and ICA (218/2920). The study population had a high number of cardiovascular risk factors, with a cumulative of 0.2 (0.4) risk factors per patient (Table 1) while the average pretest probability was 47.9% (22.2%). The prevalence of obstructive CAD in the 31 eligible studies varied between 22 and 90% (Appendix Table 8) depending on the type of patients included as well as local patient selection for ICA and conduct of functional testing (Appendix Tables 5 and 6).

### Effectiveness of CTA and functional testing for the diagnosis of obstructive CAD: individual-patient data analysis

**Fig. 3:**
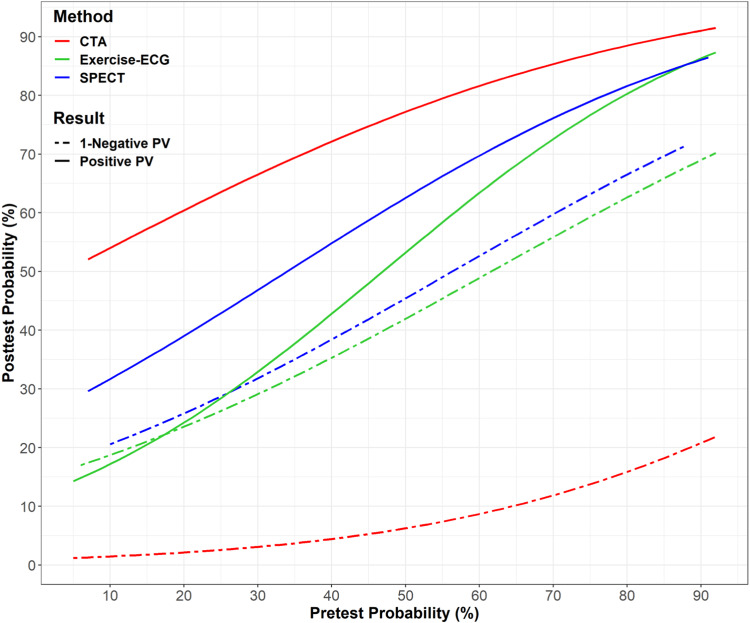
Similar diagnostic performance of CTA, Exercise-ECG, and SPECT after excluding studies with risk of bias. Similar diagnostic performance as shown in Fig. 2 after including all individual-patient data, is found in this analysis in which studies with a high risk of bias [7, 28, 33, 36, 40, 43, 44, 48] were excluded and only studies with low risk of bias were included (details on the risk of bias assessment is shown in Appendix Table 2)

For CTA, the sensitivity of 2072 patients with CT and functional test results in comparison to ICA as the reference standard in a generalised linear model was 94.6% (95% CI (92.7–96) and specificity was 76% (72.2–80) Table 2). The sensitivity of exercise-ECG was 54.9% (47.9–61.7) and specificity was 60.9% (55.2–66.3) while the sensitivity of SPECT was 72.9% (65–79.6) and specificity was 44.9% (36.7–54.4). Table 2 additionally shows all characteristics in 10%-steps of pretest probability. The sensitivity and specificity of CTA and functional stress testing differed significantly (*p* < 0.0001 for all, see Table 3). Excluding the eight studies with a high risk of bias Appendix Table 2) [7, 25, 30, 33, 37, 40, 41, 45] in a sensitivity analysis, results remained similar for all three tests (Fig. 3 and Appendix Table 11). When comparing the diagnostic accuracy of CTA in the 2920 patients included from 31 studies with functional tests performed with the 2412 patients who were included in studies without functional tests performed we found no differences indicating no relevant selection bias (Appendix Table 12).

**Table 2:** Diagnostic accuracy of CTA, exercise-ECG, and SPEC

Pre-test probability	All subjects	< 10%		10 to < 20%		20 to < 30%		30 to < 40%		40 to < 50%		50 to < 60%		60 to < 70%		70 to < 80%		80 to < 90%		90 to 100%	
*N*	2920	41		313		328		451		454		432		314		254		296		37	
TP	1424	7		85		113		198		234		213		165		171		213		25	
TN	1076	22		181		160		198		159		147		100		50		50		9	
FP	330	11		44		44		49		51		60		30		22		17		2	
FN	90	1		3		11		6		10		12		19		11		16		1	
PPV	81.2 (79.4–83)	61.1 (38.6–83.6)		65.9 (57.7–74.1)		72 (64.9–79)		80.2 (75.2–85.1)		82.1 (77.7–86.6)		78 (73.1–82.9)		84.6 (79.6–89.7)		88.6 (84.1–93.1)		92.6 (89.2–96)		92.6 (82.7–100)	
NPV	92.3 (90.7–93.8)	95.7 (87.3–100)		98.4 (96.5–100)		93.6 (89.9–97.2)		97.1 (94.7–99.4)		94.1 (90.5–97.6)		92.5 (88.3–96.6)		84 (77.5–90.6)		82 (72.3–91.6)		75.8 (65.4–86.1)		90 (71.4–100)	
Sensitivity	94.1 (92.9–95.2)	87.5 (64.6–100)		96.6 (92.8–100)		91.1 (86.1–96.1)		97.1 (94.7–99.4)		95.9 (93.4–98.4)		94.7 (91.7–97.6)		89.7 (85.3–94.1)		94 (90.5–97.4)		93 (89.7–96.3)		96.2 (88.8–100)	
Specificity	76.5 (74.3–78.7)	66.7 (50.6–82.8)		80.4 (75.3–85.6)		78.4 (72.8–84.1)		80.2 (75.2–85.1)		75.7 (69.9–81.5)		71 (64.8–77.2)		76.9 (69.7–84.2)		69.4 (58.8–80.1)		74.6 (64.2–85)		81.8 (59–100)	
LR+	4	2.6		4.9		4.2		4.9		3.9		3.3		3.9		3.1		3.7		5.3	
LR−	0.08	0.2		0.04		0.1		0.04		0.05		0.08		0.1		0.09		0.09		0.05	
*N*	1540	22		163		168		210		229		245		170		144		179		10	
TP	721	4		38		51		86		103		112		93		94		132		8	
TN	585	10		101		82		96		93		99		48		28		27		1	
FP	182	7		22		31		22		28		27		18		14		12		1	
FN	52	1		2		4		6		5		7		11		8		8			
PPV	59.8 (56.3–63.4)	66.7 (40–93.3)		70.1 (59.9–80.4)		60 (48.1–71.9)		51.6 (41.5–61.6)		56.2 (46.7–65.7)		55.4 (46.2–64.6)		72.5 (62.7–82.3)		76.6 (67.2–86.1)		91.5 (86.2–96.8)		100 (100–100)	
NPV	58.6 (55.2–62)	90 (71.4–100)		80.2 (71.8–88.6)		71.8 (63.2–80.5)		60 (51–69)		60.5 (51.9–69.1)		57.1 (48.7–65.6)		51.1 (40.8–61.4)		64.2 (52.7–75.7)		58.9 (47.6–70.2)		60 (17.1–100)	
Sensitivity	56.8 (53.3–60.3)	80 (44.9–100)		57.5 (42.2–72.8)		52.7 (39.5–65.9)		50 (39.8–60.2)		54.6 (45.2–64)		52.1 (43.1–61.1)		55.8 (46.2–65.3)		57.8 (48.3–67.4)		69.3 (61.6–76.9)		62.5 (29–96)	
Specificity	61.5 (58.1–65)	52.9 (29.2–76.7)		56.1 (47.3–64.9)		65.5 (56.7–74.3)		58.5 (49.6–67.4)		62 (53.3–70.6)		60.3 (51.8–68.9)		66.7 (55.3–78)		57.1 (42.2–72.1)		76.9 (63.7–90.1)		100 (100–100)	
LR+	1.5	1.7		1.3		1.5		1.2		1.4		1.3		1.7		1.3		3		1.7	
LR−	0.7	0.4		0.8		0.7		0.9		0.7		0.8		0.7		0.7		0.4		0.4	
*N*	532	6		63		47		60		79		74		66		49		77		11	
TP	277	1		19		17		31		38		43		34		33		53		8	
TN	166	2		33		19		20		30		16		20		9		15		2	
FP	60	2		10		9		9		8		11		6		3		2			
FN	29	1		1		2				3		4		6		4		7		1	
PPV	63.6 (58.5–68.7)	66.7 (28.9–100)		62.8 (48.3–77.2)		51.7 (33.5–69.9)		61.1 (45.2–77)		55.8 (42.3–69.3)		72.3 (59.6–85.1)		66.7 (52.4–80.9)		81.5 (66.8–96.1)		84.3 (74.3–94.3)		75 (45–100)	
NPV	53.4 (46.3–60.5)			80 (62.5–97.5)		77.8 (58.6–97)		62.5 (43.1–81.9)		55.6 (36.8–74.3)		51.9 (33–70.7)		50 (30–70)		68.2 (48.7–87.6)		65.4 (47.1–83.7)		100 (100–100)	
Sensitivity	70.9 (65.8–76)	100 (100–100)		80 (62.5–97.5)		78.9 (60.6–97.3)		71 (55–86.9)		70.7 (56.8–84.7)		72.3 (59.6–85.1)		70 (55.8–84.2)		59.5 (43.6–75.3)		71.7 (60.3–83.1)		66.7 (35.9–97.5)	
Specificity	54.9 (48.4–61.4)	100 (100–100)		62.8 (48.3–77.2)		50 (31.5–68.5)		51.7 (33.5–69.9)		60.5 (45–76.1)		51.9 (33–70.7)		53.8 (34.7–73)		58.3 (30.4–86.2)		52.9 (29.2–76.7)		100 (100–100)	
LR+	1.6			2.2		1.6		1.5		1.8		1.5		1.5		1.4		1.5		0.9	
LR−	0.5	0		0.3		0.4		0.6		0.5		0.5		0.6		0.7		0.5		0.3	

Data show the diagnostic performance for all three non-invasive tests versus invasive angiography as the reference standard with 95% CI divided into 10% steps of pre-test probability. PPV and NPV are positive and negative predictive values

*LR* likelihood ratio

**Table 3:** Overall statistical model without additional covariates

Generalised linear mixed model (basic)			
Fixed effects	Estimate (S.E.)	95% LCI	95% UCI
Intercept	−1.170 (0.110)	−1.387	−0.953
CATH yes	4.032 (0.166)	3.706	4.358
ECG	0.726 (0.103)	0.525	0.927
SPECT	1.374 (0.163)	1.054	1.694
CATH yes * ECG	−3.392 (0.173)	−3.732	−3.052
CATH yes * SPECT	−3.245 (0.244)	−3.723	−2.767

Random effects	Variance	Standard deviation	Correlations
Study no. (intercept)	0.311	0.558	
CATH yes	0.261	0.511	−0.180
Patient in study	2.125e-05	0.0046	−0.53, 0.02

Each effect is significant; fixed effects: estimates of regression coefficients. The intercept stands for 1-specificity and the sum of the intercept and CATH yes represents sensitivity. Random effects quantify between-study and between-patient variability. The variance of the random effects of the intercept corresponds to the between-studies variability of 1-specificity, the random effects variance of CATH yes to between-studies variability of sensitivity, and the random effects variance of Patient in Study corresponds to the between-patient variability within studies. As can be seen from the results in Table 3 for 1-specificity (intercept) with random effects variance equal to 0.311 and random effects variance equal to 0.265 for sensitivity (Catheter yes)

*SE* standard error, *CI* 95% confidence interval, *LCI* lower confidence interval, *UCI* upper confidence interval

There was better diagnostic differentiation using CTA compared with both exercise-ECG and SPECT (Fig. 2). Reliably excluding CAD with an NPV of 85% was possible in case of a negative CTA in patients presenting a pretest probability of up to 74% whereas negative exercise-ECG and SPECT excluded CAD only up to pretest probabilities of 7% and 11%, respectively (Fig. 2), with variability between studies (Table 3). Gender comparison showed similar results of women and men of CTA and functional tests in women and men (Appendix Tables 7, 9, 10).

### Effectiveness of CTA and functional testing for the diagnosis of obstructive CAD: study-level analysis

**Fig. 4:**
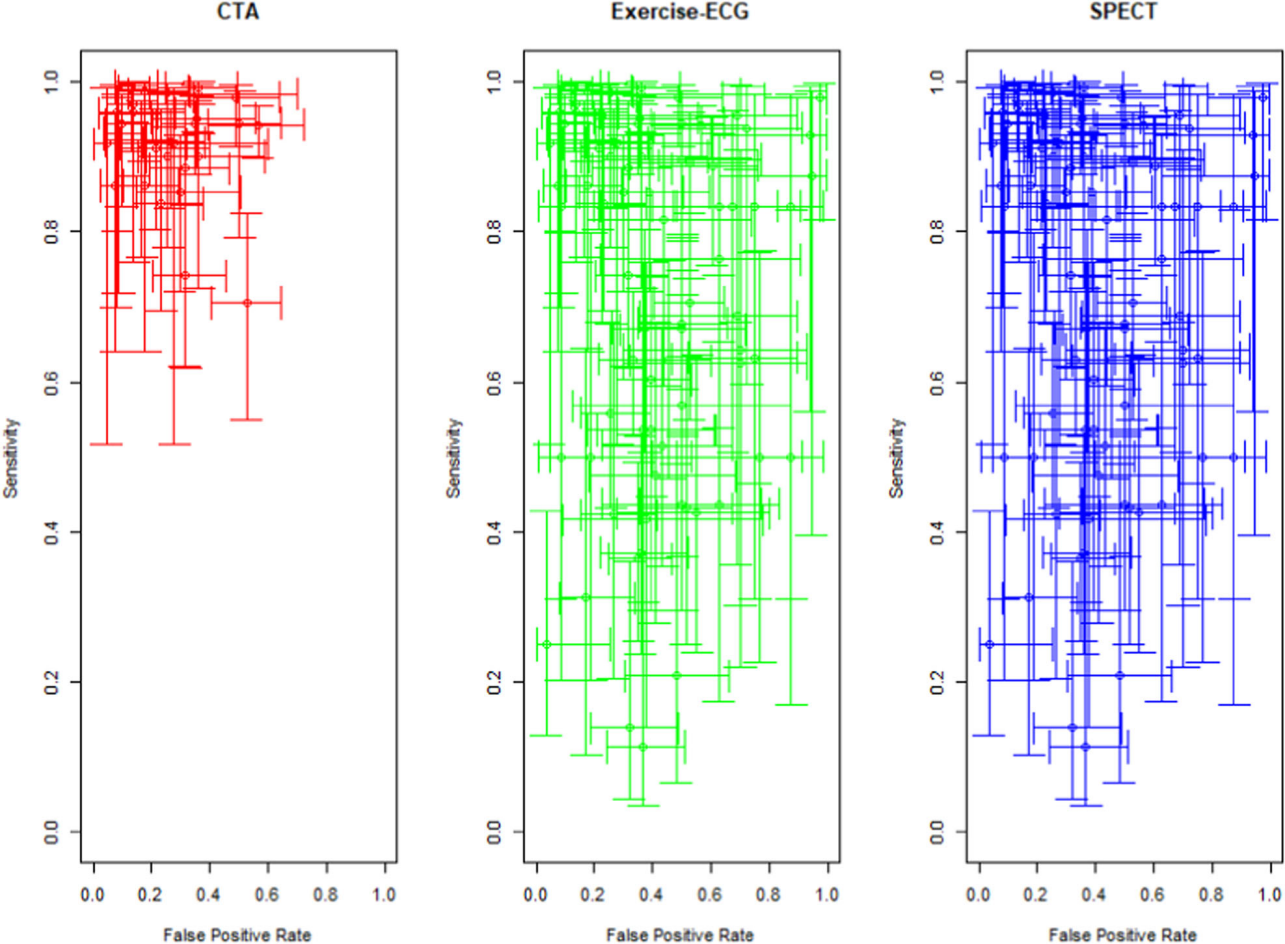
Cross-hair comparison of CTA, Exercise-ECG, SPECT of per-study sensitivity, and false-positive rate. The lines represent 95% confidence intervals for sensitivity and false-positive rate based on the per-study data for CT, exercise-ECG, and SPECT. The per-study forest plots for all three tests and the results of all individual studies are also shown in Appendix Figs. 5–8

On the study level, the sensitivity and specificity of CT, exercise-ECG, and SPECT are reported in Fig. 4. At a pretest probability of 10%, the positive predictive value (PPV) of CTA was 50.9% (95% CI 40.9–60.2) while the PPV of exercise-ECG was 19.1% (95% CI 12.8%–27.5%) and that of SPECT was 32.2 (95% CI 22.5–45.2). At a pretest probability of 74%, the NPV of CTA was 85.2% (95% CI 78.0–90.4) while the NPV of exercise-ECG was 41.9% (95% CI 32.5–0.50.6) and that of SPECT was 34.8% (95% CI 24.6–49.0).

### Multiple imputation analysis and covariates

Results of the multiple imputation analysis based on all 2920 patients (Appendix Table 7) revealed a significant influence of the covariates Agatston Score, heart rate, and chest pain on the specificity of CTA and functional test results with CTA outperforming SPECT and ECG in terms of sensitivity and specificity. Patients with an increased heart rate and higher Agatston Score with lower specificity using all diagnostic tests. The type of chest pain mainly influenced the specificity of all (functional and anatomical) diagnostic tests, which were best in patients with typical angina pectoris. Higher heart rates led to lower sensitivity of all tests. Models investigating test-specific effects of the covariates failed to converge so that only the overall influence of these covariates is reported.

## Discussion

In this pooled analysis of patient-level data, we show that both the sensitivity, as well as specificity of coronary CTA, are higher than that of exercise-ECG and SPECT for the diagnostic assessment of CAD using ICA as the reference standard. The findings are not applicable to the detection of myocardial ischaemia, which was not included in the COME-CCT protocol. Across a wide range of clinical pretest probabilities, the diagnostic performance of CTA was better than that of functional stress testing. Results were consistent across populations from 21 different sites in 16 countries suggesting that the benefit of CTA is generalisable, and that CTA should be more widely adopted in patients with suspected CAD based on stable chest pain. This adds clear evidence to previous small studies that indicated CTA might outperform functional testing for the diagnosis of obstructive CAD [5–7]. Thus, CTA may provide a more solid basis for diagnostic and treatment decision-making. However, the broad inclusion for instance of all patients with atypical or typical angina pectoris might require revision as we have shown that reliably excluding obstructive CAD by CTA (NPV of at least 85%) works best up to a clinical pretest probability of 74%. Confirming obstructive CAD based on a positive CTA yields post-test probabilities of > 75% above clinical pretest probabilities of 39% which should be considered in the decision-making.

### Comparison with previous studies

In a per-study-level meta-analysis of randomised trials, CTA compared with functional stress testing was associated with a reduced incidence of myocardial infarction [47]. This further supports the conclusions from the present IPD meta-analysis of diagnostic accuracy studies. In a network meta-analysis comparing CTA, SPECT, PET, and MRI on the per-study (not per-patient) level with ICA or fractional flow reserve (FFR) it was demonstrated that each diagnostic modality has its own optimal performance pretest probability [48]. For the choice between stress testing and coronary CTA, the ESC guideline recommends considering whether patients are suitable and if local expertise in one or the other diagnostic test is present. Nowadays local expertise is commonly present for both stress testing and CTA, while our study shows that if local expertise is available, coronary CTA should be considered as the primary test for the exclusion of obstructive CAD. Our results may help in choosing the most appropriate non-invasive test before proceeding to ICA potentially resulting in an increase of the reportedly lower diagnostic yield of invasive angiography [49]. Importantly, the ISCHEMIA trial suggests that an ischaemia detection strategy with subsequent invasive interventions may not result in improved outcomes [3]. The COME-CCT consortium used (quantitative) coronary angiography as the reference standard for the direct visualisation of coronary obstructions [8]. Considering the low uptake of invasive FFR worldwide [50], the pragmatic reference standard used in COME-CCT reflects clinical practice at the time of data collection.

In line with our results, previous results indicate that CT reduces false-positive rates compared with functional testing [51]. In contrast, compared to most previous publications, exercise-ECG and SPECT performed worse in the present study, while past meta-analyses and current guidelines report 61%–68% sensitivity and 70%–77% specificity for exercise-ECG and 73%–91% sensitivity and 48%-90% specificity for SPECT our analysis reveals a much lower diagnostic performance for the two tests, likely due to the selected population [52–59]. In the prospective multicentre PICTURE trial directly comparing SPECT and CTA with ICA with 50% lumen reduction for CAD detection as the reference standard, sensitivity was 92.0 versus 54.5% for CTA and SPECT, respectively, while specificity was 87.0% versus 78.3%. Applying a 70% lumen reduction threshold for the definition of significant CAD, CTA, and SPECT yielded similar results (sensitivity 92.6% versus 59.3% and specificity 88.9% versus 81.5%) [60]. In contrast, the COME-CCT protocol prespecified 50% coronary stenosis as the definition of obstructive CAD [8] similar to almost all studies available at the time of planning this IPD analysis [49]. Moreover, using a cut-off of 50% was assumed to not miss obstructive disease as defined [61]. Importantly, we found no evidence of selection bias in our cohort when comparing the diagnostic accuracy of CTA in the patients with and without available information on functional testing. Moreover, CTA as a non-invasive anatomical test holds an advantage regarding the evaluation of further imaging criteria, such as coronary plaque analysis, which plays an important role in further risk stratification and may be useful for the prediction of future cardiovascular events. Whereas functional tests have the advantage of functional and flow-relevant assessment of the coronary arteries.

### Quality assurance and interpretation of results

To verify if exercise-ECG and SPECT were conducted according to quality standards, participating sites reported their protocols of functional tests (Appendix Tables 2 and 3). According to these data, all SPECT examinations and the majority of exercise-ECG examinations of CAD patients were done using standardised criteria [2, 52]. Thus, a likely reason for the lower diagnostic accuracy of functional stress testing compared with CTA is that these tests cannot directly visualise obstructive disease. However, in light of the ORBITA trial, a much more comprehensive strategy for the diagnosis of CAD that includes anatomic and functional criteria will be required to improve the selection of patients who benefit from the most aggressive treatment [53]. A second aspect, that may influence reported diagnostic accuracy, is verification bias [53]. The methodologically robust inclusion of all patients with functional testing and CT prior to the reference standard most likely reduced referral bias, which cannot be entirely avoided and has been reported to lead to erroneously high diagnostic sensitivity as shown by Ladapo and co-workers [54, 55]. The solid approach of comparing CT and functional testing with the reference standard ICA used in the current collaborative meta-analysis may thus explain especially the lower sensitivity for functional testing compared to reports that were influenced by referral bias. Moreover, the reference standard used for this comparison was also a morphological imaging test (invasive catheter angiography), similar to CT, which may also explain the low diagnostic accuracy of functional tests in this analysis. In addition to that evidence is missing whether functional tests using state-of-the-art technology provide better diagnostic accuracy as most of the mentioned studies were performed a SPECT generation ago. Magnetic resonance imaging, which has shown higher diagnostic accuracy than SPECT in the CE-MARC study [54], was only rarely done as cardiac stress in patients included in COME-CCT, thus intraindividual comparison was not performed. Our results are also supported by a recent study by Patel et al, demonstrating that performing CTA first leads to the highest diagnostic yield of ICA (70%) while using functional testing leads to a lower diagnostic yield (45%) [55]. Furthermore, functional stress tests have been shown not to improve discriminative ability [56]. With our results, we have also clearly shown the ability of CTA for the identification of patients with moderate-to-severe CAD. However, especially in this patient cohort these results do not necessarily prove or evaluate the possible reduction of unnecessary ICA, which should be addressed in future studies and analyses. In summary, the present work provides further evidence for the superior diagnostic accuracy of CTA compared to exercise-ECG and SPECT.

Moreover, the SCOT-HEART and the CRESCENT trial (Calcium imaging and selective CTA in comparison to functional testing for suspected CAD), both comparing CTA with functional cardiac tests, found a reduction in cardiac events for the CTA group after a median follow-up of 1.7 years or 1.2 years [4, 57, 58]. The improvement is likely due to the change in preventive therapy regimens through CTA, such as prescription of statins, aspirin and smoking cessation, especially in the large patient group with non-obstructive CAD [58]. Interestingly, a post-hoc analysis of the SCOT-HEART trial revealed an association of exercise-ECG with revascularisation procedures and future risk of adverse coronary events, but to a lower extent than CTA while CTA also offers information about undetected CAD and improves clinical decision-making [59].

### Study limitations and strengths

Our meta-analysis had three major limitations. Fourteen studies including 1367 patients (47.8%) used CT scanners with less than 64 detector rows [5, 6, 24, 26, 30–32, 34, 35, 37, 39, 41–43, 45]. These studies contributed to the majority of the non-diagnostic test results, and because of the conservative approach that was used, this led to lower sensitivities and specificities for CTA. However, CTA still outperformed SPECT and exercise-ECG. Nowadays, the use of updated state-of-the-art technology with more than 64 detector-row CT scanners may increase diagnostic performance in general.

Assuming increased diagnostic accuracy, this would also most likely lead to improvement with less frequent non-diagnostic results and an overall reduction in radiation dose leading to more wider availability of CTA to further patients. Second, the use of obstructive CAD defined by the COME-CCT collaborators as the reference standard is not optimal for evaluating functional tests. The PACIFIC study demonstrated using FFR in ICA as a reference standard for detecting hemodynamically significant stenoses, CTA with a ≥ 50% lumen reduction as a cut-off for significant stenoses criterion performed worse than PET [62]. Yet, using obstructive CAD in ICA as the reference standard reflects clinical practice with a low adoption rate of below 10% of FFR during ICA [50]. The third limitation is the amount of missing data which was addressed by using multiple imputations for reduction of bias as described to be superior to complete case analysis even with large proportions of missing data [63, 64].

The major strength of this study is the IPD meta-analysis approach to diagnostic accuracy using GLMM, which has not been used before in comparing the diagnostic accuracy of CTA with SPECT and exercise-ECG and is generally rarely employed in diagnostic accuracy studies [65–69]. There was between-study heterogeneity for 1-specificity (intercept) and sensitivity. We assume that heterogeneity was most likely due to differences in the patient population. However, GLMM can account for some degree of heterogeneity when the study is introduced into the model as a random effect, as has been done in this IPD meta-analysis [65].

## Conclusions

Coronary CTA improves the diagnostic assessment of patients with suspected obstructive CAD based on stable chest pain when compared with functional stress testing. Diagnostic benefits of CTA over cardiac stress testing are seen across a wide range of clinical pretest probabilities and CTA should become widely adopted in patients with intermediate pretest probability.

### Supplementary information

Supplementary Material

## Data Availability

The datasets used and/or analysed during the current study are available from the corresponding author upon reasonable request.

## Abbreviations

CAD: Coronary artery disease
CCS: Chronic coronary syndrome
COME-CCT: Collaborative Meta-Analysis of Cardiac CT
CTA: Coronary computed tomography angiography
exercise-ECG: Exercise electrocardiography
FFR: Fractional flow reserve
ICA: Invasive coronary angiography
IPD: Individual patient data
NPV: Negative predictive value
PPV: Positive predictive value
QCA: Quantitative coronary analysis
SD: Standard deviation
SPECT: Single-photon emission computed tomography
