# Supplementary material for: The effectiveness of coronary computed tomography angiography and functional testing for the diagnosis of obstructive coronary artery disease: results from the individual patient data Collaborative Meta-Analysis of Cardiac CT (COME-CCT)
Source: Insights Imaging. 2024 Aug 14;15:208. doi: 10.1186/s13244-024-01702-y (PMC11324632; doi:10.1186/s13244-024-01702-y)
Supplement: Supplementary file 1 — Supplementary Material [file 13244_2024_1702_MOESM1_ESM.docx]

**The effectiveness of coronary computed tomography angiography and functional testing for the diagnosis of obstructive coronary artery disease: results from the individual patient data Collaborative Meta-analysis of Cardiac CT (COME-CCT)**

**-**

**The COME-CCT Collaborators**

Supplementary Material

Data collection and quality assurance

Authors of studies fulfilling the inclusion criteria were asked to provide patient-level data. Collected individual patient data included test results of and technical information about the non-invasive tests and invasive coronary angiography, patient characteristics, chest pain, and risk factors for CAD. Study quality was assessed by two independent investigators using the Quality Assessment of Diagnostic Accuracy Studies II (QUADAS II) tool. Discrepancies in quality assessment were resolved by consensus and Appendix Figure 1 and Appendix Tables 1-4 show the adaptation of QUADAS II items for this meta-analysis and the consensus reviewers' judgment of the methodological quality of all included studies, the study scores on each item, and the respective assessment.

Supplementary Tables

**Supplementary Table 1.** QUADAS-2* items adapted for this individual-participant meta-analysis

| DOMAIN | PATIENT SELECTION | INDEX TEST | REFERENCE STANDARD | FLOW AND TIMING |
| --- | --- | --- | --- | --- |
| Description | Describe methods of patient selection: Describe included patients (prior testing, presentation, intended use of index test and setting): | Describe the index test and how it was conducted and interpreted: | Describe the reference standard and how it was conducted and interpreted: | Describe any patients who did not receive the index test(s) and/or reference standard or who were excluded from the 2x2 table (refer to flow diagram): Describe the time interval and any interventions between index test(s) and reference standard: |
| Signalling questions (yes/no/unclear) | Was a consecutive or random sample of patients enrolled? | Were the index test results interpreted without knowledge of the results of the reference standard? | Is the reference standard likely to correctly classify the target condition? | Was there an appropriate interval between index test(s) and reference standard? |
|  | Was a case-control design avoided? | If a threshold was used, was it pre-specified? | Were the reference standard results interpreted without knowledge of the results of the index test? | Did all patients receive a reference standard? |
|  | Did the study avoid inappropriate exclusions? |  |  | Did all patients receive the same reference standard? |
|  |  |  |  | Were all patients included in the analysis? |
| Risk of bias: High/low/unclear | Could the selection of patients have introduced bias? | Could the conduct or interpretation of the index test have introduced bias? | Could the reference standard, its conduct, or its interpretation have introduced bias? | Could the patient flow have introduced bias? |
| Concerns regarding applicability: High/low/unclear | Are there concerns that the included patients do not match the review question? | Are there concerns that the index test, its conduct, or interpretation differ from the review question? | Are there concerns that the target condition as defined by the reference standard does not match the review question? |  |

*QUADAS-2 referring to following reference (applies for the whole document and is mentioned in Supplementary Table 1-4): Whiting P, Rutjes AW, Reitsma JB, Bossuyt PM, Kleijnen J. The development of QUADAS: a tool for the quality assessment of studies of diagnostic accuracy included in systematic reviews. BMC Med Res Methodol 2003; 3: 25.

**Supplementary Table 2.** QUADAS-2 analysis

|  | **Studies** | | **Risk of Bias** | | | | **Applicability Concerns** | | | **Risk of Bias** | **Applicability Concerns** |
| --- | --- | --- | --- | --- | --- | --- | --- | --- | --- | --- | --- |
| # | First author | Journal, Year | Patient Selection | Index Test | Reference Standard | Flow and Timing | Patient Selection | Index Test | Reference Standard | **Total** | **Total** |
| 1 | Alkadhi H | Eur Heart J 2008 | Low | Low | Low | Low | Low | Low | Low | **Low** | **Low** |
| 2 | Alkadhi H | Heart 2010 | Low | Low | Low | Low | Low | Low | Low | **Low** | **Low** |
| 3 | Andreini D | Cardiovasc Diabetol 2010 | Low | Low | Low | Low | Low | Low | Low | **Low** | **Low** |
| 4 | Andreini D | Circ Cardiovasc Imaging 2009 | Low | Low | Low | Low | Low | Low | Low | **Low** | **Low** |
| 5 | Andreini D | J Am Coll Cardiol 2007 | Low | Low | Low | Low | Low | Low | Low | **Low** | **Low** |
| 6 | Chen CC | Int J Cardiovasc Imaging 2011 | Low | Low | Unclear | High | Low | Low | Low | **High** | **Low** |
| 7 | Dewey M | Ann Intern Med 2006 | Low | Low | Low | Low | Low | Low | Low | **Low** | **Low** |
| 8 | Dewey M | Circulation 2009 | Low | Low | Low | Low | Low | Low | Low | **Low** | **Low** |
| 9 | Ghostine S | J Am Coll Cardiol 2006 | Low | Low | Low | Low | Low | Low | Low | **Low** | **Low** |
| 10 | Halvorsen BA | Tidsskr Nor Laegeforen 2008 | X | X | X | X | X | X | X | X | X |
| 11 | Hamdan A | JACC Cardiovasc Imaging 2011 | High | Low | Low | Low | Low | Low | Low | **High** | **Low** |
| 12 | Hausleiter J | Eur Heart J 2007 | Low | Low | Low | Low | Low | Low | Low | **Low** | **Low** |
| 13 | Jenkins SM | QJM 2012 | Low | Low | Low | High | Low | Low | Low | **Low** | **Low** |
| 14 | Kajander | Circulation 2010 | Low | Low | Low | High | Low | Low | Low | **High** | **Low** |
| 15 | Kefer J | J Am Coll Cardiol 2005 | Low | Low | Low | High | Low | Low | Low | **Low** | **Low** |
| 16 | Langer C | J Comput Assist Tomogr 2009 | Low | Low | Unclear | Low | Low | Low | Low | **Unclear** | **Low** |
| 17 | Leschka S | Heart 2008 | Low | Low | Low | Low | Low | Low | Low | **Low** | **Low** |
| 18 | Martuscelli E | Eur Heart J 2004 | Low | Low | Unclear | High | Low | Low | Low | **High** | **Low** |
| 19 | Meijboom WB | J Am Coll Cardiol 2008 | Low | Low | Low | Low | Low | Low | Low | **Low** | **Low** |
| 20 | Mendoza-Rodriguez V | The Internet Journal of Cardiology 2007 | Low | Unclear | Low | Unclear | Low | Low | Low | **Unclear** | **Low** |
| 21 | Ovrehus KA | Am J Cardiol 2010 | Low | Low | Low | Low | Low | Low | Low | **Low** | **Low** |
| 22 | Pontone G | Coron Art Dis 2007 | High | Unclear | Low | Unclear | Low | Low | Low | **Unclear** | **Low** |
| 23 | Pontone G | J Am Coll Cardiol 2009 | High | Low | Low | Low | Low | Low | Low | **High** | **Low** |
| 24 | Pontone G | Clin Radiol 2007 | Low | Unclear | Low | High | Low | Low | Low | **High** | **Low** |
| 25 | Pouleur AC | Eur Radiol 2007 | Low | Low | Low | Low | Low | Low | Low | **Low** | **Low** |
| 26 | Pouleur AC | Circ Cardiovasc Imaging 2008 | Low | Low | Low | Low | Low | Low | Low | **Low** | **Low** |
| 27 | Rixe J | Circ J 2009 | Low | Unclear | Low | Low | Low | Low | Low | **Unclear** | **Low** |
| 28 | Sato A | J Nucl Cardiol 2010 | Low | Low | Unclear | High | Low | Low | Low | **High** | **Low** |
| 29 | Shabestari AA | Am J Cardiol 2007 | High | Low | Unclear | High | Low | Low | Low | **High** | **Low** |
| 30 | Ugolini P | Can J Cardiol 2009 | Low | Low | Low | Low | Low | Low | Low | **Low** | **Low** |
| 31 | Unpublished data | Ugolini P | X | X | X | X | X | X | X | X | X |

Studies with high risk of bias were those identified using criteria explained in detail in Supplementary Table 1 above.

**Supplementary Table 3.** QUADAS-2 risk of bias summary

| RISK OF BIAS | PATIENT SELECTION | INDEX TEST | REFERENCE STANDARD | FLOW AND TIMING | TOTAL |
| --- | --- | --- | --- | --- | --- |
| Low | 25 | 25 | 24 | 19 | 17 |
| High | 4 | 0 | 0 | 8 | 8 |
| Unclear | 0 | 4 | 5 | 2 | 4 |

**Supplementary Table 4.** QUADAS-2 applicability concerns summary

| APPLICABILITY CONCERNS | PATIENT SELECTION | INDEX TEST | REFERENCE STANDARD | TOTAL |
| --- | --- | --- | --- | --- |
| Low | 29 | 29 | 29 | 29 |
| High | 0 | 0 | 0 | 0 |
| Unclear | 0 | 0 | 0 | 0 |

**Supplementary Table 5.** Site data on protocols of functional tests (SPECT)

| SPECT |  |  |  |  |  |  |  |  |
| --- | --- | --- | --- | --- | --- | --- | --- | --- |
| STUDY | **SPECT USED?** | **TYPE OF SPECT** | **TYPE OF RADIO-PHARMACEUTICAL** | **TYPE OF STRESS USED?** | **PHARMA-CEUTICAL** | **ITERATIVE RECON-STRUCTION USED?** | **TYPE OF ATTENUATION CORRECTION** | **USE OF STANDARDISED FINDINGS FOR POSITIVE SPECT** |
| 1 Alkadhi H | Yes | SPECT/CT | Technetium | Pharmaco-logical | Adenosine/ Dobutamine | No | CT-based | Yes |
| 2 Alkadhi H | Yes | SPECT/CT | Technetium | Pharmaco-logical | Adenosine/ Dobutamine | No | CT-based | Yes |
| 3 Andreini D | Yes | SPECT | Technetium | Exercise | - | No | Transmission | Yes |
| 4 Andreini D | Yes | SPECT | Technetium | Exercise | - | No | Transmission | Yes |
| 5 Andreini D | Yes | SPECT | Technetium | Exercise | - | No | Transmission | Yes |
| 6 Chen CC | Yes | SPECT | Thallium | Pharmaco-logical | Dipyridamole | No | Transmission | Yes |
| 7 Dewey M | Yes | SPECT | Technetium | Exercise only | NA | Yes | Transmission | Yes |
| 8 Dewey M | Yes | SPECT | Technetium | Exercise only | NA | Yes | Transmission | Yes |
| 9 Ghostine S* |  |  |  |  |  |  |  |  |
| 10 Halvorsen BA | No | NA | NA | NA | NA | NA | NA | NA |
| 11 Hamdan A | Yes | SPECT | Technetium | Pharmaco-logical/Exercise | Adenosine | No | Prone imaging | Yes |
| 12 Hausleiter J* |  |  |  |  |  |  |  |  |
| 13 Jenkins | No | NA | NA | NA | NA | NA | NA | NA |
| 14 Kajander* |  |  |  |  |  |  |  |  |
| 15 Kefer J | Yes | SPECT | Technetium | Exercise | NA | No | Transmission | Yes |
| 16 Langer C | Yes | SPECT | Technetium | Pharmacologic | Adenosine | No | Transmission | Yes |
| 17 Leschka S | Yes | SPECT/CT | Technetium | Pharmaco-logical | Dobutamine | No | CT-based | Yes |
| 18 Martuscelli | Yes | SPECT | Technetium | Exercise | NA | No | Transmission | Yes |
| 19 Meijboom* |  |  |  |  |  |  |  |  |
| 20 Mendoza-Rodriguez | No | NA | NA | NA | NA | NA | NA | NA |
| 21 Ovrehus* |  |  |  |  |  |  |  |  |
| 22 Pontone | No | NA | NA | NA | NA | NA | NA | NA |
| 23 Pontone | No | NA | NA | NA | NA | NA | NA | NA |
| 24 Pontone | No | NA | NA | NA | NA | NA | NA | NA |
| 25 Pouleur* |  |  |  |  |  |  |  |  |
| 26 Pouleur* |  |  |  |  |  |  |  |  |
| 27 Rixe* |  |  |  |  |  |  |  |  |
| 28 Sato | No | NA | NA | NA | NA | NA | NA | NA |
| 29 Shabestari | Yes | SPECT | Technetium | Pharmaco-logical/Exercise | NA | No | Transmission | Yes |
| 30 Ugolini | No | NA | NA | NA | NA | NA | NA | NA |
| 31 Unpublished* |  |  |  |  |  |  |  |  |

* = no detailed information about site specific functional test protocols available.

NA = not available.

**Supplementary Table 6.** Site data on protocols of functional tests (exercise ECG)

| ECG |  |  |  |  |  |  |  |  |
| --- | --- | --- | --- | --- | --- | --- | --- | --- |
| STUDY | **ECG USED?** | **TYPE OF EXERCISE** | **START VALUE (WATT)** | **DURATION OF EACH STAGE (MIN)** | **INCREMENTAL INCREASE PER STAGE (WATT)** | **USE OF STANDARDISED TERMINATION CRITERIA?** | **USE OF STANDARDISED FINDINGS FOR ‘POSITIVE’ STRESS-ECG?** | **USE OF 12-CHANNEL ECG** |
| 1 Alkadhi H | No | NA | NA | NA | NA | NA | NA | NA |
| 2 Alkadhi H | No | NA | NA | NA | NA | NA | NA | NA |
| 3 Andreini D | Yes | Bicycle | 25 | 3 | 25 | NA | NA | Yes |
| 4 Andreini D | Yes | Bicycle | 25 | 3 | 25 | NA | NA | Yes |
| 5 Andreini D | Yes | Bicycle | 25 | 3 | 25 | NA | NA | Yes |
| 6 Chen CC | Yes | NA | 25 | 3 | 25 | NA | NA | Yes |
| 7 Dewey M | Yes | Bicycle | 25 | 3 | 25 | Yes | Yes | Yes |
| 8 Dewey M | Yes | Bicycle | 25 | 3 | 25 | Yes | Yes | Yes |
| 9 Ghostine S* |  |  |  |  |  |  |  |  |
| 10 Halvorsen BA | Yes | Bicycle | 50 | 1 | 10 | Yes | Yes | Yes |
| 11 Hamdan A | Yes | Bicycle | 25 | 3 | 25 | NA | NA | Yes |
| 12 Hausleiter J* |  |  |  |  |  |  |  |  |
| 13 Jenkins | Yes | Treadmill | 5 | 3 | 2 | Yes | Yes | Yes |
| 14 Kajander* |  |  |  |  |  |  |  |  |
| 15 Kefer J | Yes | Bicycle | 25 | 3 | 25 | NA | NA | Yes |
| 16 Langer C | Yes | Bicycle | 25 | 3 | 25 | NA | NA | NA |
| 17 Leschka S | No | NA | NA | NA | NA | NA | NA | NA |
| 18 Martuscelli | Yes | Bicycle | 25 | 3 | 25 | NA | NA | NA |
| 19 Meijboom* |  |  |  |  |  |  |  |  |
| 20 Mendoza-Rodriguez | Yes | Bicycle | NA | NA | NA | Yes | Yes | Yes |
| 21 Ovrehus* |  |  |  |  |  |  |  |  |
| 22 Pontone | Yes | Bicycle | 25 | 3 | 25 | Yes | Yes | Yes |
| 23 Pontone | Yes | Bicycle | 25 | 3 | 25 | Yes | Yes | Yes |
| 24 Pontone | Yes | Bicycle | 25 | 3 | 25 | Yes | Yes | Yes |
| 25 Pouleur* |  |  |  |  |  |  |  |  |
| 26 Pouleur* |  |  |  |  |  |  |  |  |
| 27 Rixe* |  |  |  |  |  |  |  |  |
| 28 Sato | Yes | Bicycle | 25 | 3 | 25 | Yes | Yes | Yes |
| 29 Shabestari | Yes | Treadmill | 25 | 3 | 25 | NA | NA | NA |
| 30 Ugolini | Yes | Treadmill | RAMP protocol | 25-45 sec | RAMP protocol | No (according to the physicians’ assessment) | Yes | No (6-leads ECG) |
| 31 Unpublished* |  |  |  |  |  |  |  |  |

* = no detailed information about site specific functional test protocols available.

NA = not available.**Supplementary Table 7.** Model results after multiple imputation

| Generalised linear mixed model after multiple imputation (pooled results) | | | |
| --- | --- | --- | --- |
| Fixed Effects | Estimate (S.E.) | 95% LCI | 95% UCI |
| Intercept | **-0·896 (0·179)** | **-1·246** | **-0·546** |
| CATH yes | **3·863 (0·283)** | **3·308** | **4·418** |
| ECG | **0·756 (0·105)** | **0·550** | **0·962** |
| SPECT | **1·408 (0·167)** | **1·080** | **1·735** |
| Age | 0·005 (0·005) | -0·004 | 0·015 |
| Male gender | -0·002 (0·100) | -0·198 | 0·194 |
| Non-anginal | **-0·393 (0·189)** | **-0·764** | **-0·023** |
| Atypical angina | **-0·378 (0·167)** | **-0·705** | **-0·051** |
| Typical angina | **-0·522 (0·178)** | **-0·871** | **-0·174** |
| Heart rate | **0·016 (0·005)** | **0·006** | **0·026** |
| Rows (≥64) | -0·245 (0·373) | -0·976 | 0·486 |
| Agatston Score | **0·022 (0·006)** | **0·011** | **0·034** |
| CATH yes * ECG | **-3·431 (0·175)** | **-3·774** | **-3·088** |
| CATH yes * SPECT | **-3·325 (0·248)** | **-3·812** | **-2·838** |
| CATH yes * Age | -0·009 (0·008) | -0·026 | 0·007 |
| CATH yes * Male Gender | 0·116 (0·172) | -0·222 | 0·454 |
| CATH yes * Non-anginal | 0·037 (0·294) | -0·540 | 0·614 |
| CATH yes * Atypical angina | -0·035 (0·256) | -0·537 | 0·467 |
| CATH yes * typical angina | 0·306 (0·261) | -0·205 | 0·818 |
| CATH yes * Heart Rate | **-0·018 (0·008)** | **-0·034** | **-0·002** |
| CATH yes * Rows (≥64) | -0·143 (0·468) | -1·062 | 0·775 |
| CATH yes * Agatston | -0·007 (0·008) | -0·023 | 0·008 |
| Random Effects | Variance | Standard deviation | Correlations |
| Study No. (Intercept) | 0·361 | 0·600 |  |
| CATH yes | 0·260 | 0·510 | -0·298 |
| Patient in Study | 1.9765e-05 | 0·0044 | -0·593 0·109 |

S.E. = standard error, CI = 95% Confidence interval; fixed effects: estimates of all regression coefficients. Positive results of the covariates decrease specificity (more false positives), negative results increase specificity, and significant results are marked bold. Interaction terms influence sensitivity. Negative values imply a reduction of sensitivity. The variance of the random effects quantifies the variability between studies for sensitivity and specificity. The variable “CATH yes” describes the invasive coronary angiography result 1= positive. The variable “CATH yes*X” describes the interaction between invasive coronary angiography results and a corresponding covariate X (e.g. gender or heart rate). Heart rate and Agatston score were entered as continuous covariates into the model. The interactions are needed to maintain the bivariate structure of the diagnostic accuracy data. Random effects quantify between-study and between-patient variability. The variance of the random effects of the intercept corresponds to the between-studies variability of 1-specificity, the random effects variance of CATH yes to between-studies variability of sensitivity; the random effects variance of Patient in Study corresponds to the between-patient variability. Pooled variances and standard deviations are derived from mean of the imputation estimates. Estimates were averaged over m=20 imputation steps and given together with the multiple imputation based variance estimates. Three way interactions, i.e. whether the covariates have a different effect on the results of the respective functional test would be desirable. The respective models failed to converge and thus no meaningful results are available. The results present the best fitting model. Age and gender did not influence the results significantly. However, when adjusting for these covariates, we still obtained similar results for the diagnostic performance of the respective methods (see also table 3): that is, compared to CTA, SPECT and ECG show lower sensitivity and specificity.

**Supplementary Table 8.** Detailed characteristics per study

| STUDY | YEAR | NR, OF PTS. | WOMEN (%) | AGE  (MEAN ± SD) | CAD BY ICA | CT | ECG | SPECT | PTP (MEAN ± SD) | TYPE OF PATIENTS INCLUDED |
| --- | --- | --- | --- | --- | --- | --- | --- | --- | --- | --- |
| All IPD datasets |  | 2920 | 963 (33%) | 61 (±10) | 1514 (52%) | 2920 (100%) | 1540 (53%) | 532 (18%) | 48% (±22%) |  |
| 1 Alkadhi H | 2008 | 150 | 47 (31%) | 64 (±12) | 59 (39%) | 150 (100%) | 54 (36%) | 13 (9%) | 51 (±22%) | “referred to ICA for clinical reasons”, “stable clinical conditions” |
| 2 Alkadhi H | 2010 | 99 | 26 (26%) | 63 (±8) | 35(35%) | 99 (100%) | 65 (66%) | 11 (11%) | 47% (±21%) | “patients with low-to-intermediate risk and suspicion of coronary artery disease” |
| 3 Andreini D | 2010 | 210 | 26 (12%) | 61 (±8) | 180 (86%) | 210 (100%) | 47 (22%) | 21 (10%) | 47% (±19%) | “patients […]with indication to ICA for suspected CAD” |
| 4 Andreini D | 2009 | 127 | 48 (38%) | 56 (±7) | 46 (36%) | 127 (100%) | 31 (24%) | 14 (11%) | 26% (±12%) | “patients […] affected by DCM […] of unknown etiology”  “Exclusion criteria included […], a history of CAD, […]” |
| 5 Andreini D | 2007 | 170 | 49 (29%) | 54 (±8) | 84 (49%) | 170 (100%) | 47 (28%) | 29 (17%) | 28% (±13%) | “61 unknown origin DCM patients […] and 139 patients with normal cardiac function with indications for coronary angiography”  Only a small part of the control group without DCM presented with known CAD, this is one reason why only a subgroup of the patients from this study was included in the present analysis as only patients with stable chest pain and without known CAD were available for inclusion in our Consortium. |
| 6 Chen CC | 2011 | 75 | 14 (19%) | 61 (±10) | 50 (67%) | 75 (100%) | 28 (37%) | 50 (67%) | 58% (±19%) | “symptomatic patients with chest pain or chest discomfort referred for cardiac CT scan, including CS and coronary angiography. Patients underwent CCA in all cases, regardless of the CTA results” |
| 7 Dewey M | 2006 | 29 | 20 (69%) | 60 (±10) | 11 (38%) | 29 (100%) | 18 (62%) | 7 (24%) | 36% (±21%) | “patients with suspected coronary artery disease” |
| 8 Dewey M | 2009 | 129 | 95 (74%) | 63 (±9) | 67 (52%) | 129 (100%) | 80 (62%) | 38 (29%) | 45% (±22%) | “patients with suspected coronary artery disease referred for clinically indicated conventional coronary angiography” |
| 9 Ghostine S | 2006 | 32 | 17 (53%) | 70 (±13) | 15 (47%) | 32 (100%) | 0 (0%) | 7 (22%) | 64% (±22%) | “patients with complete LBBB, without a history of CAD, admitted for CCA” |
| 10 Halvorsen BA | 2008 | 83 | 37 (45%) | 61 (±9) | 39 (47%) | 83 (100%) | 82 (99%) | 3 (4%) | 56% (±21%) | “patients with suspected stabile coronary disease, referred to invasive coronary angiography” |
| 11 Hamdan A | 2011 | 88 | 32 (36%) | 64 (±9) | 44 (50%) | 88 (100%) | 17 (19%) | 15 (17%) | 50% (±22%) | “patients with suspected or known coronary artery disease prospectively underwent 32-channel 3.0-T MRI and 64-slice CT before elective X-ray angiography” |
| 12 Hausleiter J | 2007 | 243 | 85 (35%) | 62 (±10) | 101 (42%) | 243 (100%) | 127 (52%) | 10 (4%) | 45% (±20%) | “patients with an intermediate pre-test probability for having CAD and who were scheduled to have elective invasive coronary angiography” |
| 13 Jenkins | 2011 | 99 | 44 (44%) | 58 (±11) | 38 (38%) | 99 (100%) | 91 (92%) | 30 (30%) | 49% (±27%) | “Inclusion criteria were suspected cardiac ischaemia on the basis of symptoms and non-invasive stress testing with subsequent referral for elective I-CA to determine the presence or absence of CAD” |
| 14 Kajander | 2010 | 99 | 43 (43%) | 64 (±7) | 43 (43%) | 99 (100%) | 89 (90%) | 0 (0%) | 58% (±22%) | “patients with an intermediate (30% to 70%) pretest likelihood of coronary artery disease” |
| 15 Kefer J | 2005 | 41 | 9 (22%) | 64 (±13) | 24 (59%) | 41 (100%) | 31 (76%) | 25 (61%) | 59% (±26%) | “patients […] who were referred […] for conventional diagnostic X-ray coronary angiography were enrolled […]. Indications for cardiac catheterization were: typical angina and positive stress test in 35 patients, atypical chest pain or dyspnea with positive stress test in 6 patients, silent ischemia in 3 patients, and chest pain with negative stress test in 6 patients. Five patients were referred to evaluate coronary anatomy before non coronary cardiac surgery and one patient because of ventricular tachycardia” |
| 16 Langer C | 2009 | 68 | 30 (44%) | 64 (±11) | 26 (38%) | 68 (100%) | 36 (53%) | 13 (19%) | 44% (±17%) | “patients […] presenting atypical angina […].”  “patients referred to our institution by a cardiologist for elective CA” |
| 17 Leschka S | 2008 | 67 | 23 (34%) | 61 (±12) | 29 (43%) | 67 (100%) | 11 (16%) | 0 (0%) | 55% (±23%) | “patients […] who were scheduled for CCA because of typical […] or atypical chest pain […], pathological exercise test […] or dyspnoea” |
| 18 Martuscelli | 2004 | 64 | 5 (8%) | 59 (±7) | 43 (67%) | 64 (100%) | 64 (100%) | 0 (0%) | 61% (±20%) | “patients […] with suspected coronary artery disease” |
| 19 Meijboom | 2008 | 108 | 33 (31%) | 61 (±6) | 69 (64%) | 108 (100%) | 67 (62%) | 50 (46%) | 57% (±21%) | “symptomatic patients who presented with stable anginal syndromes and unstable anginal syndromes who were referred for clinically indicated CCA” |
| 20 Mendoza-Rodriguez | 2009 | 81 | 20 (25%) | 56 (±8) | 18 (22%) | 81 (100%) | 60 (74%) | 22 (27%) | 51% (±22%) | “patients in whom their coronary tree was first studied by MSCT and afterwards by ICA” |
| 21 Ovrehus | 2010 | 100 | 50 (50%) | 61 (±9) | 29 (29%) | 100 (100%) | 100 (100%) | 0 (0%) | 43% (±23%) | “patients referred for invasive coronary angiography […] because of suspicion of CAD” |
| 22 Pontone | 2007 | 144 | 35 (24%) | 62 (±10) | 95 (66%) | 144 (100%) | 144 (100%) | 0 (0%) | 59% (±19%) | “patients who had already performed an exercise electrocardiogram test referred to our hospital and scheduled for coronary angiography for chest pain” |
| 23 Pontone | 2009 | 160 | 24 (15%) | 65 (±10) | 144 (90%) | 160 (100%) | 35 (22%) | 53 (33%) | 60% (±18%) | “patients […] scheduled for ICA for suspected CAD” |
| 24 Pontone | 2007 | 116 | 30 (26%) | 63 (±10) | 63 (54%) | 116 (100%) | 43 (37%) | 0 (0%) | 43% (±16%) | “patients with suspected CAD, scheduled for conventional coronary angiography” |
| 25 Pouleur | 2008 | 75 | 19 (25%) | 60 (±13) | 17 (23%) | 75 (100%) | 38 (51%) | 8 (11%) | 40% (±20%) | “patients […] scheduled to undergo valve surgery underwent 40-slice MDCT before invasive quantitative coronary angiography” |
| 26 Pouleur | 2007 | 15 | 6 (40%) | 62 (±12) | 5 (33%) | 15 (100%) | 7 (47%) | 3 (20%) | 39% (±21%) | “patients referred to our institution for conventional diagnostic x-ray coronary angiography […] who were in sinus rhythm and who had no prior revascularization procedure (no stent or bypass operation)” |
| 27 Rixe | 2009 | 76 | 29 (38%) | 63 (±10) | 40 (53%) | 76 (100%) | 72 (95%) | 0 (0%) | 49% (±21%) | “patients […] referred for invasive coronary angiography (CAG) because of suspected CAD” |
| 28 Sato | 2010 | 107 | 38 (36%) | 67 (±10) | 59 (55%) | 107 (100%) | 0 (0%) | 107 (100%) | 64% (±22%) | “symptomatic patients with suspected CAD underwent both 64-slice CTA and stress thallium-201 MPI before invasive coronary angiography” |
| 29 Shabestari | 2007 | 113 | 32 (28%) | 64 (±10) | 90 (80%) | 113 (100%) | 62 (55%) | 27 (24%) | 36% (±15%) | “patients who under-went both coronary MSCT and ICA in a single institution were studied in a prospective manner. Their assessment indications were suspicion for CAD (based on either clinical presentation in the form of stable angina or unstable angina pectoris and/or equivocal exercise tolerance test/myocardial perfusion radionuclide study results in patients presenting with atypical chest pain) or their schedule for coronary artery bypass grafting.” |
| 30 Ugolini | 2009 | 13 | 5 (13%) | 62 (±5) | 5 (38%) | 13 (100%) | 5 (38%) | 0 (0%) | 56% (±28%) | “Patients […] scheduled for clinically indicated, nonurgent coronary angiography” |
| 31 Unpublished | N/A | 3* | 1 (33%) | 54 (±16) | 0 (0%) | 3 (100%) | 1 (33%) | 0 (0%) | 43% (±17%) | No information available |

SD = standard deviation, N/A = not available, PTP = Pre-test probability, Nr. of pts = number of patients

* This unpublished study was included despite only providing 3 patients for this comparison of CT with functional tests as the entire study #31 included 15 patients.

**Supplementary Table 9.** Overall statistical model with gender as an additional covariate

|  | | | | | | | | | | |
| --- | --- | --- | --- | --- | --- | --- | --- | --- | --- | --- |
| Fixed Effects | | | Estimate (S.E.) | | | Z value | | | P value | |
| Intercept | | | **-1·14967 (0·12395)** | | | **-9·275** | | | **<0·001** | |
| Gender | | | -0·03444 (0·09655) | | | -0·357 | | | 0·721 | |
| Cath | | | **3·89153 (0·20083)** | | | **19·377** | | | **<0·001** | |
| Method1 | | | **0·72557 (0·10250)** | | | **7·079** | | | **<0·001** | |
| Method2 | | | **1·37244 (0·16298)** | | | **8·421** | | | **<0·001** | |
| Gender:Cath | | | 0·19807 (0·16737) | | | 1·183 | | | 0·237 | |
| Cath:Method1 | | | **-3·39504 (0·17308)** | | | **-19·616** | | | **<0·001** | |
| Cath:Method2 | | | **-3·24037 (0·24329)** | | | **-13·319** | | | **<0·001** | |
| Correlation of fixed effects | | | | | | | | | | |
|  | (Intr) | Gender | | Cath | Method1 | | Method2 | Gender:Cath | | Cath:Method1 |
| Gender | -0.454 |  | |  |  | |  |  | |  |
| Cath | -0.375 | 0.271 | |  |  | |  |  | |  |
| Method1 | -0.332 | 0.006 | | 0.205 |  | |  |  | |  |
| Method2 | -0.229 | 0.018 | | 0.142 | 0.227 | |  |  | |  |
| Gender:Cath | 0.252 | -0.557 | | -0.561 | -0.005 | | -0.010 |  | |  |
| Cath:Method1 | 0.199 | -0.004 | | -0.484 | -0.588 | | -0.136 | -0.023 | |  |
| Cath:Method2 | 0.153 | -0.011 | | -0.344 | -0.156 | | -0.661 | 0.009 | | 0.360 |
| Random Effects | | | Variance | | | Standard deviation | | | Correlations | |
| Study No. (Intercept) | | | 0·361 | | | 0·600 | | |  | |
| CATH yes | | | 0·260 | | | 0·510 | | | -0·298 | |
| Patient in Study | | | 1.9765e-05 | | | 0·0044 | | | -0·593 0·109 | |

This is the overall statistical model (as shown in Table 3) yet including gender as a covariate.

**Supplementary Table 10.** Comparison of sensitivity and specificity stratified in the model for gender and diagnostic procedure.

|  | | | | | |
| --- | --- | --- | --- | --- | --- |
| Gender analysis | | | | | |
|  | **Probe** | **Standard error** | **Df** | **Lower 95%-CI** | **Upper 95%-CI** |
| Method = 0, gender = 0 | | | | | |
| 1-specificity | 0·241 | 0·02264 | Inf | 0·199 | 0·288 |
| Sensitivity | 0·939 | 0·01094 | Inf | 0·914 | 0·958 |
| Method = 1, gender = 0 | | | | | |
| 1-specificity | 0·396 | 0·03156 | Inf | 0·336 | 0·459 |
| Sensitivity | 0·518 | 0·04430 | Inf | 0·432 | 0·604 |
| Method = 2, gender = 0 | | | | | |
| 1-specificity | 0·555 | 0·04463 | Inf | 0·467 | 0·640 |
| Sensitivity | 0·706 | 0·04454 | Inf | 0·612 | 0·785 |
| Method = 0, gender = 1 | | | | | |
| 1-specificity | 0·234 | 0·02109 | Inf | 0·196 | 0·278 |
| Sensitivity | 0·948 | 0·00834 | Inf | 0·929 | 0·962 |
| Method = 1, gender = 1 | | | | | |
| 1-specificity | 0·387 | 0·03002 | Inf | 0·330 | 0·448 |
| Sensitivity | 0·559 | 0·03632 | Inf | 0·487 | 0·628 |
| Method = 2, gender = 1 | | | | | |
| 1-specificity | 0·547 | 0·04412 | Inf | 0·460 | 0·631 |
| Sensitivity | 0·738 | 0·03762 | Inf | 0·658 | 0·805 |

There were similar results in women and men and gender did not significantly influence model results.

**Supplementary Table 11.** Comparison of sensitivity and specificity stratified in the model for studies with and without high risk of bias and diagnostic procedure.

|  | | | | | |
| --- | --- | --- | --- | --- | --- |
| Studies without high risk of bias in QUADAS | | | | | |
|  | **Probe** | **Standard error** | **Df** | **Lower 95%-CI** | **Upper 95%-CI** |
| CT | | | | | |
| 1-specificity | 0·223 | 0·02210 | Inf | 0·183 | 0·269 |
| Sensitivity | 0·956 | 0·00864 | Inf | 0·936 | 0·971 |
| ECG | | | | | |
| 1-specificity | 0·385 | 0·03209 | Inf | 0·325 | 0·450 |
| Sensitivity | 0·516 | 0·04246 | Inf | 0·433 | 0·598 |
| SPECT | | | | | |
| 1-specificity | 0·600 | 0·05175 | Inf | 0·496 | 0·696 |
| Sensitivity | 0·710 | 0·04897 | Inf | 0·606 | 0·796 |

There were similar results based on studies with and without high risk of bias.

**Supplementary Table 12.** Model based on studies with data on functional testing versus studies without functional testing data.

|  | | | | |
| --- | --- | --- | --- | --- |
| Model based on studies with vs. without data on functional testing | | | | |
|  | **Estimate** | **Standard error** | **Z value** | **Pr(>\|z\|)** |
| (Intercept) | -1.363 | 0.193 | -7.049 | 0 |
| Cath_pos | 4.273 | 0.453 | 9.432 | 0 |
| studyin | 0.046 | 0.25 | 0.183 | 0.855 |
| Cath_pos:studyin | 0.059 | 0.575 | 0.102 | 0.919 |

Likelihood Ratio Test 0.12, df=2, p=0.94

|  |
| --- |

|  |
| --- |

|  |
| --- |

|  |
| --- |

Supplementary Figures


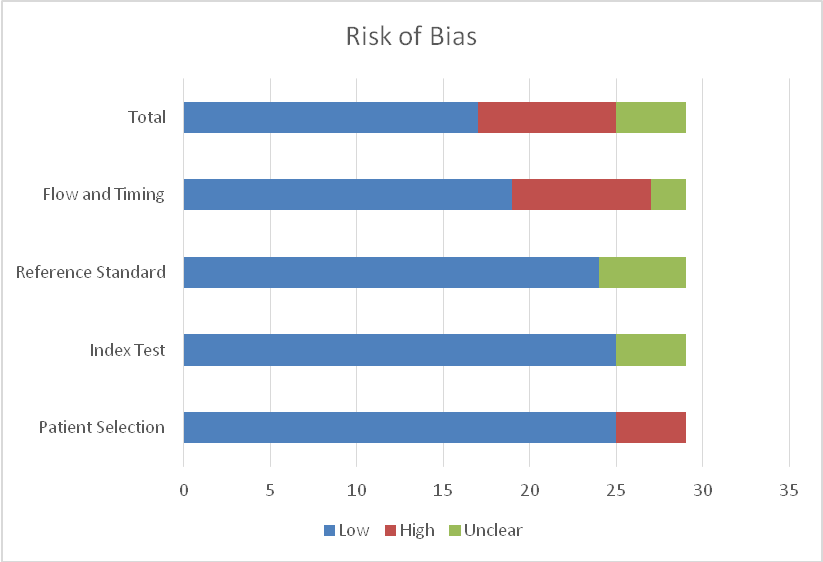


***Supplementary Figure 1:* Consensus reviewers’ judgment of the methodological quality of included studies regarding risk of bias**

Quality was assessed using the QUADAS 2 tool by two reviewers. In case of disagreement adjudication with a third reviewer was performed. The proportional rate of studies (in %) with a low (blue), high (red) or unclear (green) risk of bias is displayed on the X-axis. The corresponding items under investigation are displayed on the Y-axis.


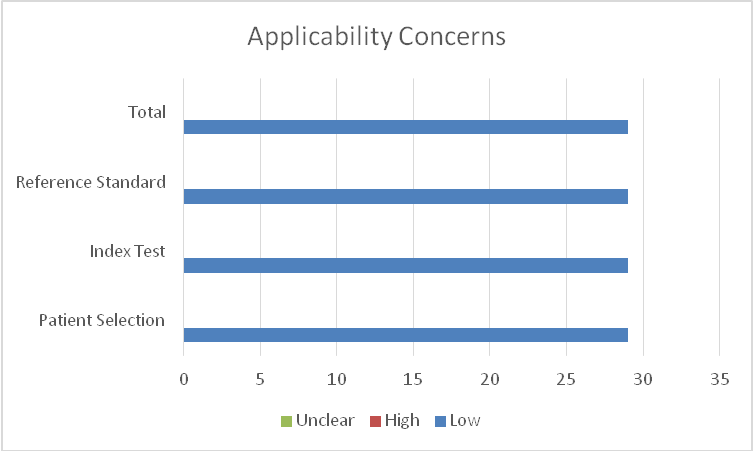


***Supplementary Figure 2:* Consensus reviewers’ judgment of the methodological quality of included studies regarding applicability concerns**

The X-axis displays the proportional rate of studies (in %) bearing a low (blue), high (red) or unclear (green) risk of bias regarding the items displayed on the Y-axis. There are only low concerns regarding the applicability of studies included in the analysis.


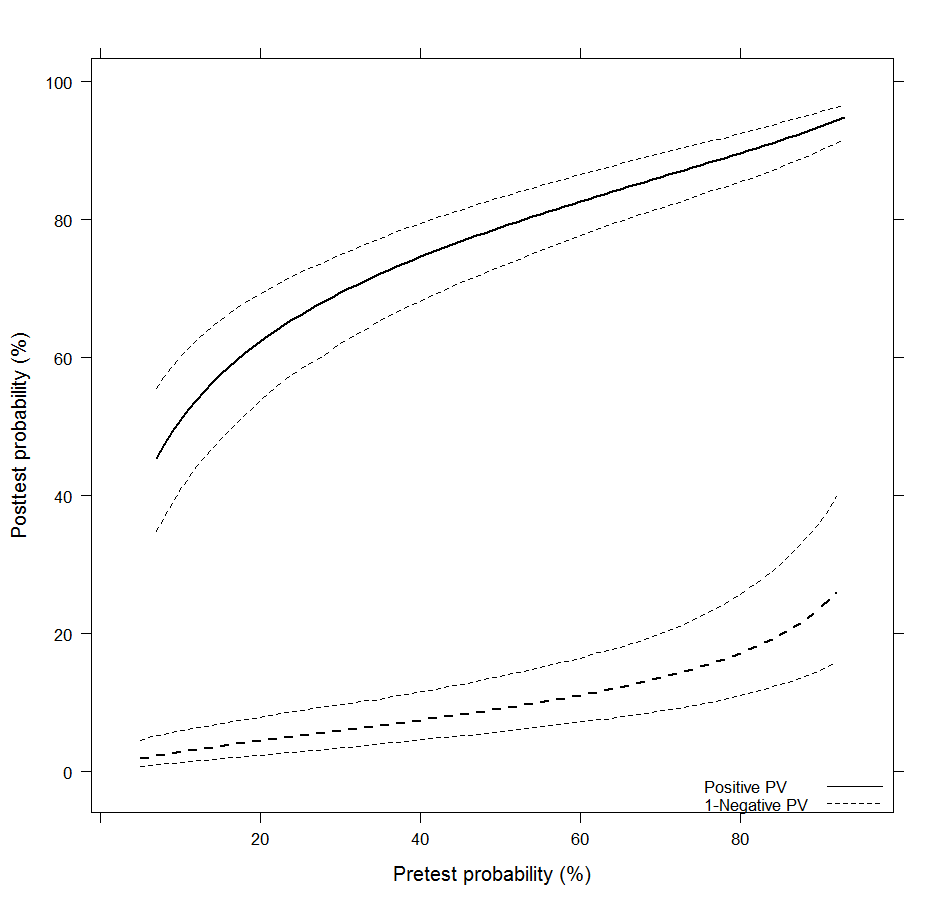


***Supplementary Figure 3:*** **Analysis of diagnostic performance for CTA**

Positive and negative predictive values of CAD (with surrounding 95% confidence interval) after a positive (solid lines) or negative (dashed lines) diagnostic test result for obstructive (angiographically-significant) coronary artery disease defined as a patient with at least 50% coronary diameter stenosis.


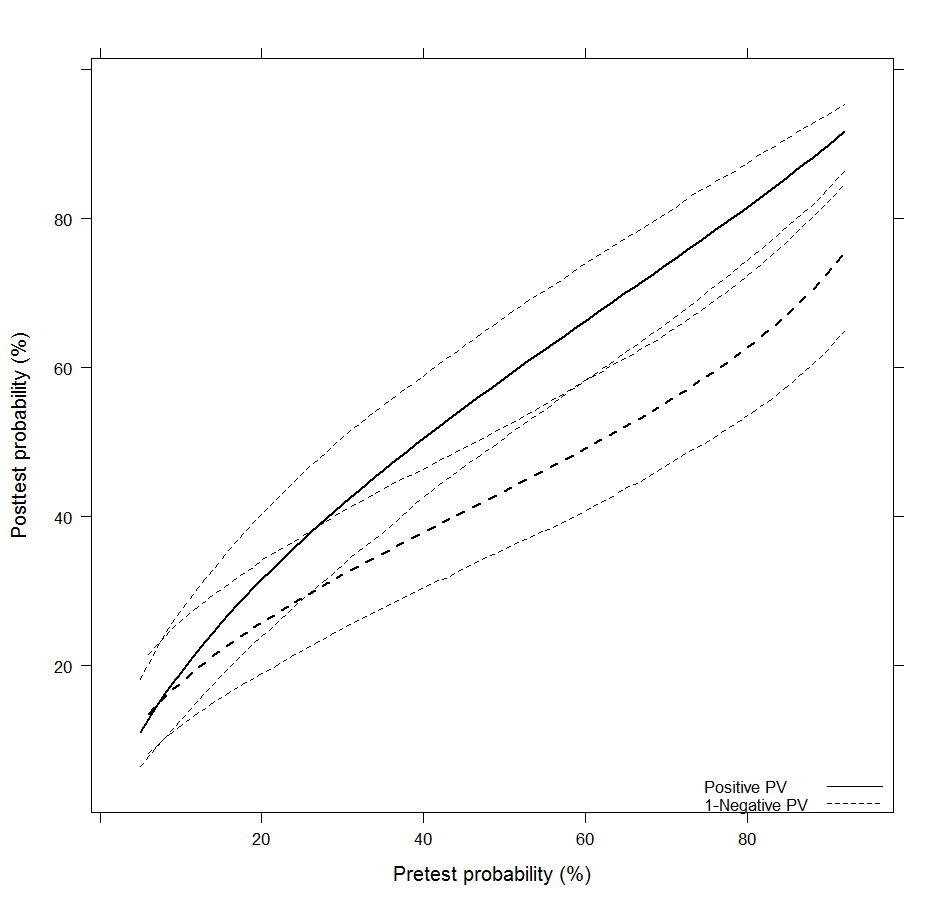


***Supplementary Figure 4:*** **Analysis of diagnostic performance for exercise ECG**

Positive and negative predictive values of CAD (with surrounding 95% confidence interval) after a positive (solid lines) or negative (dashed lines) diagnostic test result for obstructive (angiographically-significant) coronary artery disease defined as a patient with at least 50% coronary diameter stenosis.


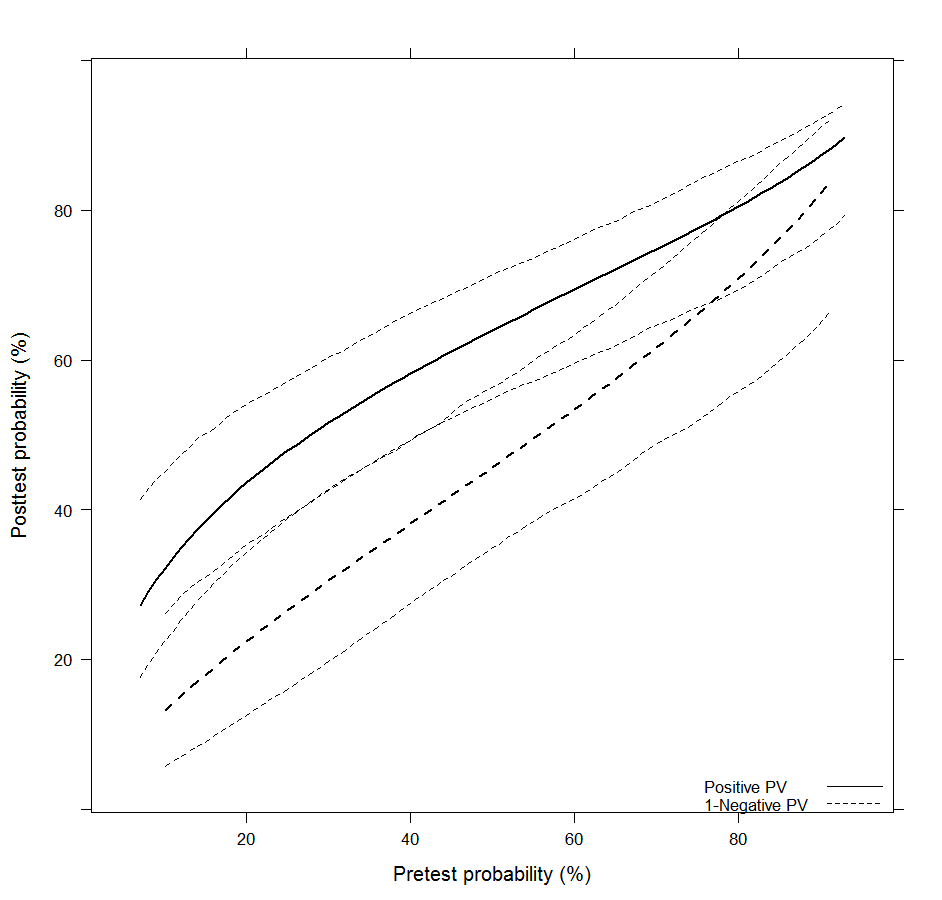


***Supplementary Figure 5:*** **Analysis of diagnostic performance for SPECT**

Positive and negative predictive values of CAD (with surrounding 95% confidence interval) after a positive (solid lines) or negative (dashed lines) diagnostic test result for obstructive (angiographically-significant) coronary artery disease defined as a patient with at least 50% coronary diameter stenosis.


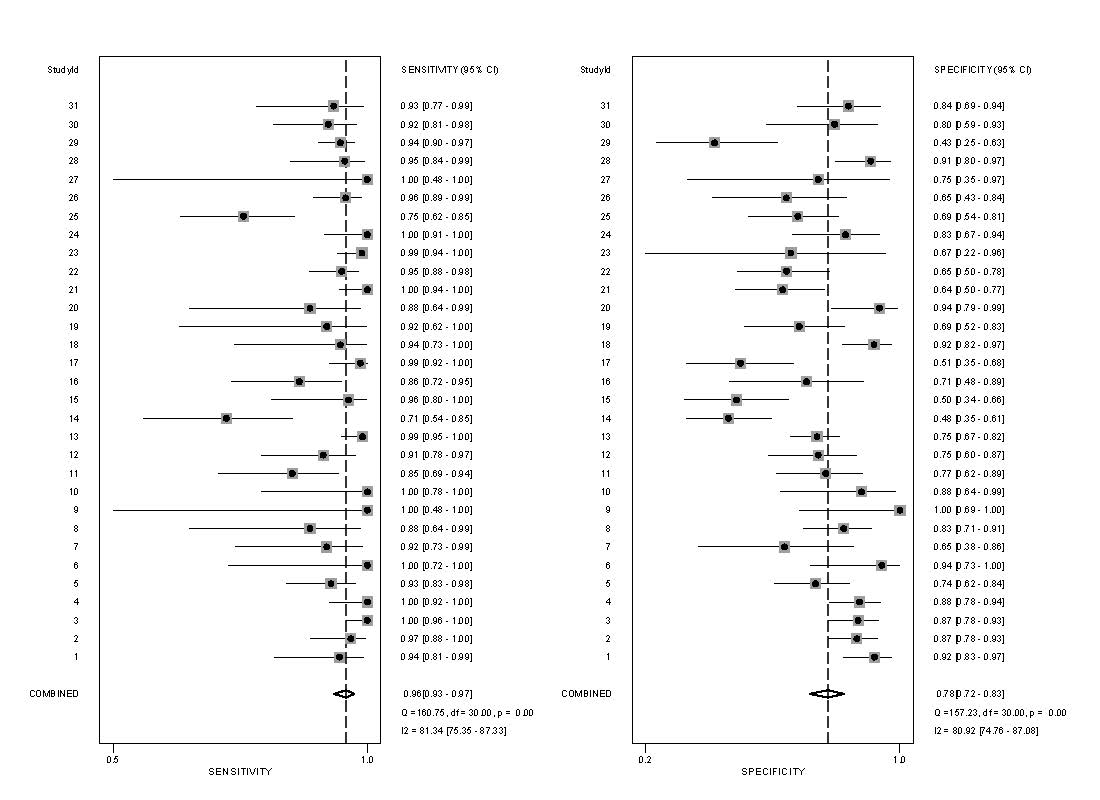


***Supplementary Figure 6:*** **Forest plot of all included studies for CTA**

Sensitivity and specificity are shown with 95% confidence intervals for each study included in this meta-analysis for CTA.

Supplementary References of included studies

**References of studies included in this individual patient data (IPD) meta-analysis**

1. Alkadhi H, Scheffel H, Desbiolles L, et al. Dual-source computed tomography coronary angiography: influence of obesity, calcium load, and heart rate on diagnostic accuracy. *Eur Heart J* 2008;29(6):766-76. doi: 10.1093/eurheartj/ehn044 [published Online First: 2008/02/23]

2. Alkadhi H, Stolzmann P, Desbiolles L, et al. Low-dose, 128-slice, dual-source CT coronary angiography: accuracy and radiation dose of the high-pitch and the step-and-shoot mode. *Heart* 2010;96(12):933-8. doi: 10.1136/hrt.2009.189100 [published Online First: 2010/06/12]

3. Andreini D, Pontone G, Bartorelli AL, et al. Comparison of the diagnostic performance of 64-slice computed tomography coronary angiography in diabetic and non-diabetic patients with suspected coronary artery disease. *Cardiovasc Diabetol* 2010;9:80. doi: 10.1186/1475-2840-9-80 [published Online First: 2010/12/01]

4. Andreini D, Pontone G, Bartorelli AL, et al. Sixty-four-slice multidetector computed tomography: an accurate imaging modality for the evaluation of coronary arteries in dilated cardiomyopathy of unknown etiology. *Circ Cardiovasc Imaging* 2009;2(3):199-205. doi: 10.1161/CIRCIMAGING.108.822809 [published Online First: 2009/10/08]

5. Andreini D, Pontone G, Pepi M, et al. Diagnostic accuracy of multidetector computed tomography coronary angiography in patients with dilated cardiomyopathy. *J Am Coll Cardiol* 2007;49(20):2044-50. doi: 10.1016/j.jacc.2007.01.086 [published Online First: 2007/05/22]

6. Chen CC, Chen CC, Hsieh IC, et al. The effect of calcium score on the diagnostic accuracy of coronary computed tomography angiography. *Int J Cardiovasc Imaging* 2011;27 Suppl 1:37-42. doi: 10.1007/s10554-011-9955-6 [published Online First: 2011/10/14]

7. Dewey M, Teige F, Schnapauff D, et al. Noninvasive detection of coronary artery stenoses with multislice computed tomography or magnetic resonance imaging. *Ann Intern Med* 2006;145(6):407-15. doi: 10.7326/0003-4819-145-6-200609190-00004 [published Online First: 2006/09/20]

8. Dewey M, Zimmermann E, Deissenrieder F, et al. Noninvasive coronary angiography by 320-row computed tomography with lower radiation exposure and maintained diagnostic accuracy: comparison of results with cardiac catheterization in a head-to-head pilot investigation. *Circulation* 2009;120(10):867-75. doi: 10.1161/CIRCULATIONAHA.109.859280 [published Online First: 2009/08/26]

9. Ghostine S, Caussin C, Daoud B, et al. Non-invasive detection of coronary artery disease in patients with left bundle branch block using 64-slice computed tomography. *J Am Coll Cardiol* 2006;48(10):1929-34. doi: 10.1016/j.jacc.2006.04.103 [published Online First: 2006/11/23]

10. Halvorsen BA, Rodevand O, Hagen G, et al. [Angiography with 64-channel CT upon suspicion of stable coronary disease]. *Tidsskr Nor Laegeforen* 2008;128(19):2172-6. [published Online First: 2008/10/11]

11. Hamdan A, Asbach P, Wellnhofer E, et al. A prospective study for comparison of MR and CT imaging for detection of coronary artery stenosis. *JACC Cardiovasc Imaging* 2011;4(1):50-61. doi: 10.1016/j.jcmg.2010.10.007 [published Online First: 2011/01/15]

12. Hausleiter J, Meyer T, Hadamitzky M, et al. Non-invasive coronary computed tomographic angiography for patients with suspected coronary artery disease: the Coronary Angiography by Computed Tomography with the Use of a Submillimeter resolution (CACTUS) trial. *Eur Heart J* 2007;28(24):3034-41. doi: 10.1093/eurheartj/ehm150 [published Online First: 2007/06/02]

13. Jenkins SM, Johnston N, Hawkins NM, et al. Limited clinical utility of CT coronary angiography in a district hospital setting. *QJM* 2011;104(1):49-57. doi: 10.1093/qjmed/hcq163 [published Online First: 2010/09/18]

14. Kajander S, Joutsiniemi E, Saraste M, et al. Cardiac positron emission tomography/computed tomography imaging accurately detects anatomically and functionally significant coronary artery disease. *Circulation* 2010;122(6):603-13. doi: 10.1161/CIRCULATIONAHA.109.915009 [published Online First: 2010/07/28]

15. Kefer J, Coche E, Legros G, et al. Head-to-head comparison of three-dimensional navigator-gated magnetic resonance imaging and 16-slice computed tomography to detect coronary artery stenosis in patients. *J Am Coll Cardiol* 2005;46(1):92-100. doi: 10.1016/j.jacc.2005.03.057 [published Online First: 2005/07/05]

16. Langer C, Peterschroder A, Franzke K, et al. Noninvasive coronary angiography focusing on calcification: multislice computed tomography compared with magnetic resonance imaging. *J Comput Assist Tomogr* 2009;33(2):179-85. doi: 10.1097/RCT.0b013e3181839624 [published Online First: 2009/04/07]

17. Leschka S, Scheffel H, Desbiolles L, et al. Combining dual-source computed tomography coronary angiography and calcium scoring: added value for the assessment of coronary artery disease. *Heart* 2008;94(9):1154-61. doi: 10.1136/hrt.2007.124800 [published Online First: 2007/11/23]

18. Martuscelli E, Romagnoli A, D'Eliseo A, et al. Accuracy of thin-slice computed tomography in the detection of coronary stenoses. *Eur Heart J* 2004;25(12):1043-8. doi: 10.1016/j.ehj.2004.03.024 [published Online First: 2004/06/12]

19. Meijboom WB, Meijs MF, Schuijf JD, et al. Diagnostic accuracy of 64-slice computed tomography coronary angiography: a prospective, multicenter, multivendor study. *J Am Coll Cardiol* 2008;52(25):2135-44. doi: 10.1016/j.jacc.2008.08.058 [published Online First: 2008/12/20]

20. Mendoza V, Llerena L, Llerena L, et al. Ischemic heart disease diagnosed by 64 slice computed tomography coronary angiography. *The Internet Journal of Cardiology* 2009;7(1)

21. Ovrehus KA, Jensen JK, Mickley HF, et al. Comparison of usefulness of exercise testing versus coronary computed tomographic angiography for evaluation of patients suspected of having coronary artery disease. *Am J Cardiol* 2010;105(6):773-9. doi: 10.1016/j.amjcard.2009.11.006 [published Online First: 2010/03/10]

22. Pontone G, Andreini D, Ballerini G, et al. Diagnostic work-up of unselected patients with suspected coronary artery disease: complementary role of multidetector computed tomography, symptoms and electrocardiogram stress test. *Coron Artery Dis* 2007;18(4):265-74. doi: 10.1097/MCA.0b013e328035f8ae [published Online First: 2007/05/15]

23. Pontone G, Andreini D, Bartorelli AL, et al. Diagnostic accuracy of coronary computed tomography angiography: a comparison between prospective and retrospective electrocardiogram triggering. *J Am Coll Cardiol* 2009;54(4):346-55. doi: 10.1016/j.jacc.2009.04.027 [published Online First: 2009/07/18]

24. Pontone G, Andreini D, Quaglia C, et al. Accuracy of multidetector spiral computed tomography in detecting significant coronary stenosis in patient populations with differing pre-test probabilities of disease. *Clin Radiol* 2007;62(10):978-85. doi: 10.1016/j.crad.2007.02.022 [published Online First: 2007/09/04]

25. Pouleur AC, le Polain de Waroux JB, Kefer J, et al. Usefulness of 40-slice multidetector row computed tomography to detect coronary disease in patients prior to cardiac valve surgery. *Eur Radiol* 2007;17(12):3199-207. doi: 10.1007/s00330-007-0676-0 [published Online First: 2007/06/06]

26. Pouleur AC, le Polain de Waroux JB, Kefer J, et al. Direct comparison of whole-heart navigator-gated magnetic resonance coronary angiography and 40- and 64-slice multidetector row computed tomography to detect the coronary artery stenosis in patients scheduled for conventional coronary angiography. *Circ Cardiovasc Imaging* 2008;1(2):114-21. doi: 10.1161/CIRCIMAGING.107.756304 [published Online First: 2009/10/08]

27. Rixe J, Rolf A, Conradi G, et al. Detection of relevant coronary artery disease using dual-source computed tomography in a high probability patient series: comparison with invasive angiography. *Circ J* 2009;73(2):316-22. doi: 10.1253/circj.cj-08-0534 [published Online First: 2009/01/06]

28. Sato A, Nozato T, Hikita H, et al. Incremental value of combining 64-slice computed tomography angiography with stress nuclear myocardial perfusion imaging to improve noninvasive detection of coronary artery disease. *J Nucl Cardiol* 2010;17(1):19-26. doi: 10.1007/s12350-009-9150-5 [published Online First: 2009/09/25]

29. Shabestari AA, Abdi S, Akhlaghpoor S, et al. Diagnostic performance of 64-channel multislice computed tomography in assessment of significant coronary artery disease in symptomatic subjects. *Am J Cardiol* 2007;99(12):1656-61. doi: 10.1016/j.amjcard.2007.01.040 [published Online First: 2007/06/15]

30. Ugolini P, Pressacco J, Lesperance J, et al. Evaluation of coronary atheroma by 64-slice multidetector computed tomography: Comparison with intravascular ultrasound and angiography. *Can J Cardiol* 2009;25(11):641-7. doi: 10.1016/s0828-282x(09)70161-8 [published Online First: 2009/11/10]
